# Cell-Permeant and Photocleavable Chemical Inducer of Dimerization**

**DOI:** 10.1002/anie.201310969

**Published:** 2014-03-26

**Authors:** Mirjam Zimmermann, Ruben Cal, Elia Janett, Viktor Hoffmann, Christian G Bochet, Edwin Constable, Florent Beaufils, Matthias P Wymann

**Affiliations:** University of Basel, Department of BiomedicineMattenstrasse 28, Basel (Switzerland); University of Fribourg, Department of ChemistryChemin du Musée 9, Fribourg (Switzerland); University of Basel, Department of ChemistrySpitalstrasse 51, Basel (Switzerland)

**Keywords:** chemical inducers of dimerization, fusion proteins, photocleavable linkers, photolysis, protein–protein interactions

## Abstract

Chemical inducers of dimerization (CIDs) have been developed to orchestrate protein dimerization and translocation. Here we present a novel photocleavable HaloTag- and SNAP-tag-reactive CID (MeNV-HaXS) with excellent selectivity and intracellular reactivity. Excitation at 360 nm cleaves the methyl-6-nitroveratryl core of MeNV-HaXS. MeNV-HaXS covalently links HaloTag- and SNAP-tag fusion proteins, and enables targeting of selected membranes and intracellular organelles. MeNV-HaXS-mediated translocation has been validated for plasma membrane, late endosomes, lysosomes, Golgi, mitochondria, and the actin cytoskeleton. Photocleavage of MeNV-HaXS liberates target proteins and provides access to optical manipulation of protein relocation with high spatiotemporal and subcellular precision. MeNV-HaXS supports kinetic studies of protein dynamics and the manipulation of subcellular enzyme activities, which is exemplified for Golgi-targeted cargo and the assessment of nuclear import kinetics.

Localization of signaling enzymes is key to controlling protein and lipid kinase cascades in physiology and disease.[1] Control of protein localization and enzyme activity by illumination provides unique access to the manipulation of biological processes in living cells with high spatiotemporal precision. Caged small molecules and enzyme substrates have been developed for a number of applications.[2]

Naturally occurring light-sensitive protein domains have been used to design genetically encoded light-controlled protein–protein interaction modules. These so-called optogenetic systems contain a photoisomerizable chromophore, which undergoes a conformational change upon illumination at a defined wavelength. Optogenetic systems have been used to control the activation of single signaling proteins by protein caging (light-inducible GTPase Rac),[3] or in a more modular approach to indirectly manipulate cellular signaling, through the light-dependent dimerization of two protein modules.[4] Optogenetic light-activated dimerization systems are versatile tools, but suffer from several drawbacks such as large photosensory protein tags,[4,5] the requirement of exogenous cofactors,[4] slow kinetics,[5] formation of unwanted homodimers,[6] and sensitivity to environmental light, and/or overlap with excitation wavelength of popular fluorescent reporter proteins.[6,7] Another approach to control protein localization and enzyme activity are chemical inducers of dimerization (CIDs)[8] and self-localizing ligands,[9] which have been successfully used to manipulate signaling pathways including phosphoinositide turnover,[10] and small GTPases.[11]

Presently, cell-permeable CIDs that can be efficiently manipulated intracellularly have not been reported.[8] Some spatial selectivity has been achieved with photocleavable, biotinylated α-methylnitrobenzylrapamycin, which has been used to control small GTPase activity.[12] This caged rapamycin was targeted to an extracellular location by means of its biotin moiety, required, however, extracellular photolytic removal of the caging group before rapamycin was released to diffuse across the cell membrane.[12] Another photocaged rapamycin derivative is pRap.[13] Both of these noncovalent, photocleavable CIDs provide a source of highly diffusible dimerizer, limiting local target manipulation.

Here we present a novel photocleavable CID, which forms a covalent link between HaloTag-[14] and SNAP-tag[15]-fused proteins. The photocleavable methyl-6-nitroveratryl (MeNV) group was introduced into the core module linking the HaloTag-reactive chloroalkane ligand and the SNAP-tag-reactive O6-benzylguanine, and the cell permeability of the resulting CID molecule is retained (dubbed MeNV-HaXS; Figure 1). The combination of chemical-induced dimerization and the possibility of a subsequent light-induced reversal of the protein–protein interaction combines the advantage of a modular approach of genetically encodable tags with a highly specific spatiotemporal control by light.

**Figure 1:**
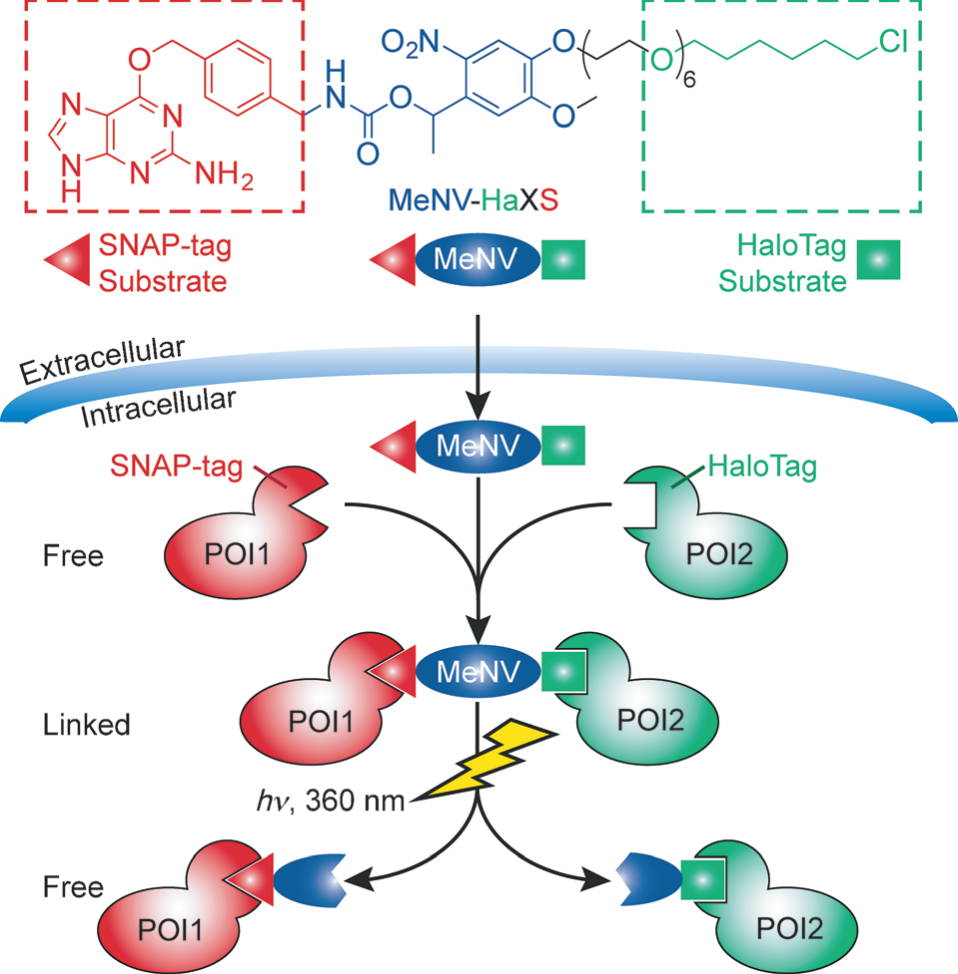
A photocleavable, cell-permeable HaloTag- and SNAP-tag-reactive CID with a methyl-6-nitroveratryl (MeNV) core was generated (MeNV-HaXS). After cell entry, MeNV-HaXS dimerizes HaloTag- and SNAP-tag-fused proteins of interest (POI). Illumination of MeNV-HaXS (360 nm; *ε*=4058 m^−1^ cm^−1^; quantum yield=0.075) cleaves the link between the POIs, and releases them from the covalent complex. For the synthesis of MeNV-HaXS see the Supporting Information.

As depicted schematically in Figure 1, MeNV-HaXS penetrates cells and induces the dimerization of HaloTag and SNAP-Tag fusion proteins to form a covalently stabilized complex. Upon illumination (360 nm), the MeNV group undergoes photolysis, which triggers the cleavage of the protein dimer and the release of cargo proteins.

MeNV-HaXS was optimized to match the cell permeability of the noncleavable dimerizer of HaloTag and SNAP-tag fusion proteins called HaXS8.[16] Time-dependent dimerization of HaloTag-GFP and SNAP-tag-GFP fusion proteins expressed in HeLa cells was studied in response to the addition of MeNV-HaXS and HaXS8, and we found that MeNV-HaXS and HaXS8 produced dimers at a similar rates. Slight differences were measurable at earlier time points (*t*<10 min, Figure 2 a), which became insignificant after 15 min of treatment. These slight differences may be explained by the increased molecular weight and higher polarity of MeNV-HaXS, resulting from the incorporation of the PEG6 element. MeNV-HaXS-induced HaloTag-SNAP-tag dimers were stable for >5 h, and exposure to ambient light did not affect the stability of dimers.

**Figure 2:**
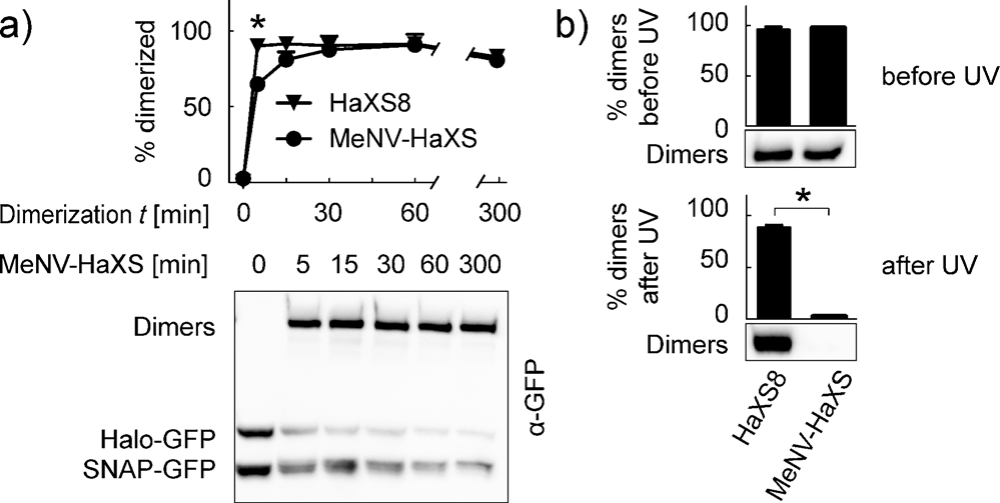
MeNV-HaXS induces the formation of intracellular dimers of HaloTag and SNAP-tag fusion proteins, which are cleaved upon UV illumination. a) HeLa cells transfected with expression constructs for SNAP-tag-GFP (SNAP-GFP) and HaloTag-GFP (Halo-GFP) fusion proteins were exposed to 5 μm MeNV-HaXS or 5 μm light-insensitive HaXS8 for the indicated times in cell culture medium at 37 °C. Subsequently, cells were lysed and proteins were subjected to SDS-PAGE and immunoblotting. Tagged proteins were detected using anti-GFP (primary) and horseradish peroxidase labeled (secondary) antibodies, and chemiluminescence (means±SEM, *n*=3). b) HeLa cells expressing SNAP-GFP and Halo-GFP as in (a) were incubated with 5 μm MeNV-HaXS or HaXS8 for 15 min. Cells were then washed and submerged in phosphate-buffered saline (PBS) to remove unreacted compounds, and illuminated with a high-intensity UV lamp (100 W, 5 cm distance) for 10 min. Analysis of dimerization products was performed as in (a); values represent means±SEM, *n*=3; * indicates *p*<0.05.

Due to the matched dimerization properties of MeNV-HaXS and HaXS8, the noncleavable HaXS8 is a valuable control compound to monitor the efficiency of photocleavage, and to detect potential side effects exerted by UV irradiation. The efficiency of the intracellular photocleavage of MeNV-HaXS was tested in HeLa cells expressing HaloTag-GFP and SNAP-tag-GFP fusion proteins. A HaloTag-SNAP-tag complex preformed with MeNV-HaXS could be cleaved in bulk by illumination with a 360 nm lamp (Blak-Ray, B-100A, UVP). Quantification of protein dimers before and after exposure to UV light revealed that MeNV-HaXS-induced dimers were quantitatively cleaved after 10 min of illumination, whereas HaXS8-containing dimers remained intact (Figure 2 b).

MeNV-HaXS thus offers the possibility to trigger covalent dimerization, and to subsequently release associated proteins in a controlled way. This allows the manipulation of protein localization, which can be exploited to mimic cellular signaling events in a timed and localized fashion. Many signaling events take place at defined intracellular locations. The combination of a tagged anchor protein and MeNV-HaXS exposure permits the depletion of tagged signaling enzymes from their productive sites. Presently, MeNV-HaXS has been validated for the targeting of tagged proteins to intracellular organelles such as Golgi (Figure 3 a), plasma membrane, lysosomes, mitochondria, and the actin skeleton (Figure S2).

**Figure 3:**
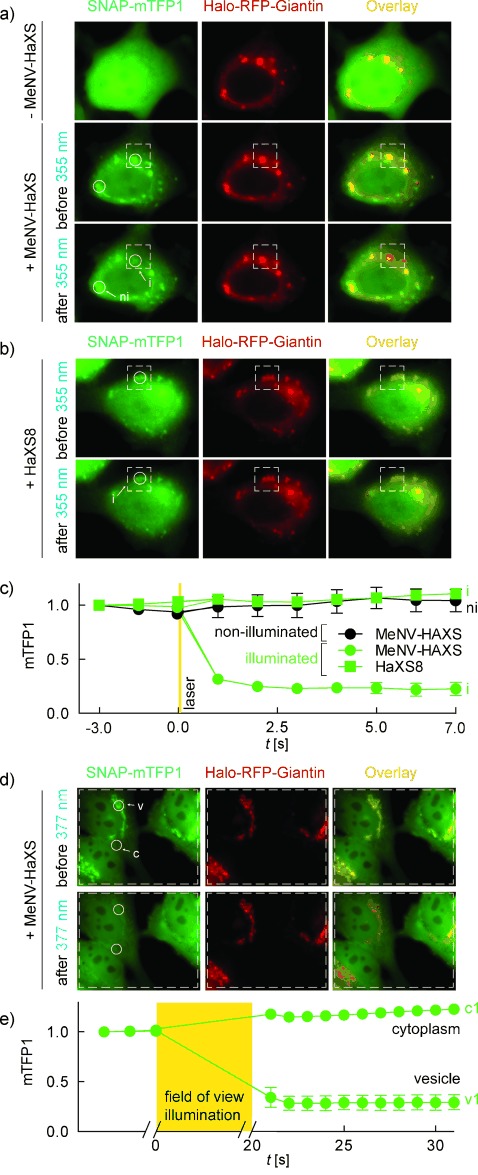
Translocation of cytosolic SNAP-mTFP1 proteins to the Golgi followed by their release upon UV illumination. a,b) HeLa cells expressing SNAP-mTFP1 and Halo-RFP-Giantin were exposed to a) 5 μm MeNV-HaXS or b) 5 μm HaXS8 in cell culture medium for 15 min at 37 °C, both of which induced translocation of cytosolic SNAP-mTFP1 to the Golgi. SNAP-mTFP1 intensity was monitored in the indicated circular regions of interest by live-cell microscopy, before and after illumination of a subcellular region within the cell (white, dotted square) with a scanning FRAP laser (8 areas × 5 ms at 355 nm). c) Quantification of mTFP1 fluorescence intensity in selected regions of interest (labeled circles) for SNAP-mTFP1 after addition of MeNV-HaXS (circles) or HaXS8 (squares). Quantifications of mTFP1 intensity in illuminated areas (green curves) and non-illuminated Golgi vesicles (black curve); values are means ±SEM, *n*=10, error bars not shown when smaller than symbols. d) HeLa cells as in (a) were exposed to 5 μm MeNV-HaXS and illuminated for 20 s using a standard DAPI filter set on a conventional fluorescence microscope (*t*=20 s, 377±25 nm). e) Quantification of mTFP1 fluorescence intensity in selected regions of interest (circles) at Golgi-derived vesicles (v), and in the cytoplasm (c) before and after illumination as described in (d); values are means ±SEM, *n*=10, error omitted when smaller than symbols; for more controls see Figure S3.

Targeted irradiation of MeNV-HaXS-anchored protein complexes using a microscope equipped with an XY scanning excitation laser for FRAP (fluorescence recovery after photobleaching; 355 nm) indeed released tagged proteins from the illuminated spots. HeLa cells were cotransfected with cytosolic teal fluorescent (cyan) SNAP-tag fusion protein (SNAP-mTFP1) and the Golgi anchor Halo-RFP-Giantin, which was constructed by the fusion of a red fluorescent protein (monomeric RFP; TagRFP) to a HaloTag and a C-terminal Golgi-targeting motif derived from giantin.[17]

Incubation with 5 μm of MeNV-HaXS or 5 μm of HaXS8 efficiently translocated cytosolic SNAP-mTFP1 to the cytosolic surface of the Golgi membrane (Figure 3 a). After an illumination pulse (8×5 ms at 355 nm) of a subset of Golgi-derived vesicles, SNAP-mTFP1 was efficiently and selectively released from illuminated, but not from the non-illuminated vesicular compartments (Figure 3 a,c). No significant loss of fluorescence intensity was observed when the light-insensitive HaXS8 was used to anchor SNAP-mTFP1 at Golgi membranes (Figure 3 b,c), confirming that the observed light-triggered decrease in fluorescence of SNAP-mTFP1 tethered with MeNV-HaXS results from the release, and not from the photobleaching of SNAP-mTFP1 fluorescence. Moreover, membrane-anchored SNAP-mTFP1 detaches rapidly from membranes after a laser pulse (*t*<1 s). This demonstrates that MeNV-HaXS can be utilized to manipulate protein localization with excellent subcellular precision on a timescale of seconds.

As a scanning FRAP laser is not part of standard fluorescence microscope equipment, we explored the possibility of using global field of view illumination with standard DAPI excitation filters (377±25 nm; standard mercury halide lamp) to induce the photocleavage of MeNV-HaXS-induced protein complexes. We found that an illumination time of <20 s was sufficient to completely liberate SNAP-mTFP1 from Golgi membranes (Figure 3 d,e). A corresponding increase of mTFP1 fluorescence intensity in the cytoplasm demonstrates that the light-induced fluorescence decrease of SNAP-mTFP1 at vesicles results from the release and not from global photobleaching of SNAP-mTFP1 fluorescence.

Although slower due to the limited excitation energy, photocleavage using DAPI excitation filters on a conventional fluorescence microscope greatly expands the range of applications offered by the MeNV-HaXS system.

In contrast to previously reported CIDs, MeNV-HaXS can instantaneously release its cargo when illuminated. To further evaluate this concept, a nuclear probe was forced to dock on a Golgi anchor. Expressed alone in HeLa cells, the nuclear localization sequence (NLS)-containing cyan fluorescent probe (NLS-CFP-SNAP) accumulated in the nucleus. In the presence of co-expressed Golgi anchor (Giantin-RFP-Halo) and added MeNV-HaXS, NLS-CFP-SNAP was trapped at perinuclear sites on the Golgi (Figure 4 a). Subsequent irradiation of cells triggered the release of NLS-CFP-SNAP from the Golgi to the cytosol within seconds (Figure 4 b). Nuclear import of liberated NLS-CFP-SNAP was delayed (Figure 4 b), reflecting nuclear import processes.[18] This illustrates that photocleavage of MeNV-HaXS is rapidly achieved, and permits the study of nuclear import kinetics in real time in a simple experimental setup.

**Figure 4:**
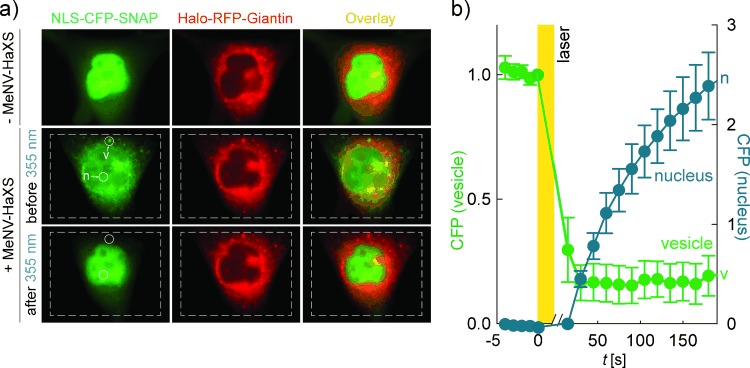
Control of nuclear export and import by MeNV-HaXS. Translocation of the NLS-CFP-SNAP probe from the nucleus to the Golgi was controlled by MeNV-HaXS and reversed by subsequent UV illumination. a) HeLa cells expressing Halo-RFP-Giantin and NLS-CFP-SNAP were exposed to 5 μm MeNV-HaXS in cell-culture medium for 15 min at 37 °C, which induced export of NLS-CFP-SNAP from the nucleus to the Golgi. CFP fluorescence intensity was monitored in the indicated circular regions of interest by live-cell microscopy, before and after illumination of the cell (white, dashed rectangle) with a scanning FRAP laser (150 areas × 5 ms at 355 nm). b) Quantification of CFP fluorescence intensity in selected regions of interest (labeled circles) monitoring vesicle-associated (green curve) and nuclear NLS-CFP-SNAP concentrations (blue curve) before and after illumination of the cells are shown; values are means ±SEM, *n*=7 cells, error bars not shown where smaller than symbols used.

In summary, MeNV-HaXS is the first cell-permeant CID giving rise to the formation of covalently linked and photocleavable protein complexes. Like other CID approaches, the design of fusion proteins bearing interacting functional protein domains must be carefully considered. The covalent link formed by MeNV-HaXS simplifies monitoring and validation of protein complexes greatly. MeNV-HaXS is therefore a novel valuable tool that combines intracellular protein dimerization with photocleavage triggered by a wavelength compatible with widely used fluorescent reporter proteins. The possibility to control protein localization by two independent events can be exploited to sequester any protein of interest away from its functional compartment. CID-dependent trapping of enzymes to nonfunctional sites has been established as an elegant approach to interrupt signaling pathways.[19,20] Optically guided cleavage of MeNV-HaXS can release anchored proteins and restore their function. Many more scenarios are possible, for example the deployment of on-off-on and off-on-off protocols and the orthogonal use of MeNV-HaXS with other CIDs. The choice between global and local illumination triggering dissociation of the CID-mediated complex opens a wide range of applications, such as investigation of cell-compartment-associated signaling, and the simulation of cell-wide physiological and pathological signaling dynamics.
