# Supplementary material for: Cell-Permeant and Photocleavable Chemical Inducer of Dimerization
Source: Angew Chem Int Ed Engl. 2014 Mar 26;53(18):4717–20. doi: 10.1002/anie.201310969 (PMC4499241; doi:10.1002/anie.201310969)

Angewandte Chemie
Eine Zeitschrift der Gesellschaft Deutscher Chemiker

Supporting Information
© Wiley-VCH 2014
69451 Weinheim, Germany

Cell-Permeant and Photocleavable Chemical Inducer of Dimerization**

Mirjam Zimmermann, Ruben Cal, Elia Janett, Viktor Hoffmann, Christian G. Bochet, Edwin Constable, Florent Beaufils, and Matthias P. Wymann**

anie_201310969_sm_miscellaneous_information.pdf

Table of Contents

1. Structure of key compound used in this study page 2

2. Supplementary experimental procedures and conditions page 3

3. Chemical synthesis and characterization of compounds page 6

4. Supplementary experiments and data page 9

5. Spectra of intermediates and MeNV-HaXS
    a. NMR spectra (^{1}H NMR and ^{13}C NMR) page 14
    b. HRMS spectra page 22

1. Structures of key compounds used in this study

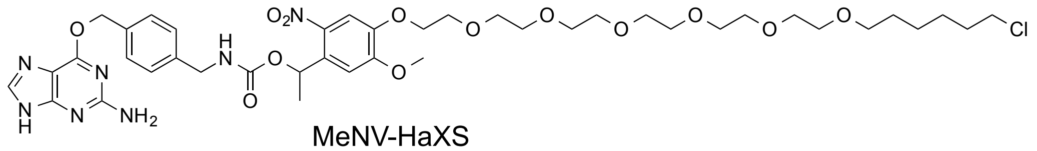

MeNV-HaXS

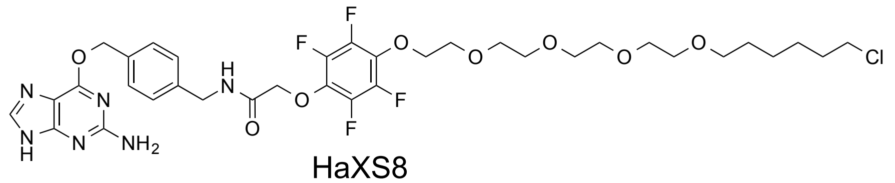

HaXS8

MeNV-HaXS (top):

1-(4-((24-chloro-3,6,9,12,15,18-hexaoxatetracosyl)oxy)-5-methoxy-2-nitrophenyl)ethyl (4-(((2-amino-9H-purin-6-yl)oxy)methyl)benzyl)carbamate

HaXS8 (bottom):

N-(4-(((2-amino-9*H*-purin-6-yl)oxy)methyl)benzyl)-2-(4-((18-chloro-3,6,9,12-tetraoxaoctadecyl)oxy)-2,3,5,6-tetrafluorophenoxy)acetamide (see reference [16] in main text).

2. Supplementary experimental procedures and conditions

Chemical synthesis and characterization

MeNV-HaXS was synthesized in 8 steps starting from acetovanillone, hexaethylene glycol, O6-aminomethylbenzylguanine and 6-chloro-1-iodohexane with good overall yield (>800 mg produced). To balance cell permeability and solubility in water, a PEG6 element was introduced. The methyl-6-nitroveratryl group (MeNV) was selected as a photocleavable group because it absorbs light at >360 nm. This minimizes cell damage, matches initiation of photocleavage to widely available excitation equipment and filters, and MeNV provides a sufficient quantum yield for intracellular photocleavage. Last but not least, intermediates and by-products emerging from the photolytic process do not interfere with living cells. The photophysical properties of MeNV-HaXS were determined *in vitro* as described below. MeNV-HaXS is not sensitive to ambient light, displays excellent cleavage efficiency at 360 nm with an absorption coefficient of 4058 $M^{-1}cm^{-1}$, and a quantum yield of 0.075. No special precautions concerning light exposure were required during synthesis.

Materials and reagents were of the highest commercially available grade and used without further purification. Reactions were monitored by thin layer chromatography (TLC) using Merck silica gel 60 F254 plates. Compounds were visualized by UV, ceric ammonium molybdate (CAM), $KMnO_4$ and ninhydrin. Flash chromatography was performed using Merck silica gel 60, particle size 40 - 63 µm.

^{1}H, ^{19}F and ^{13}C NMR spectra were recorded on a Bruker AV-400 or a DRX-600 NMR spectrometer. Chemical shifts are reported in ppm using the solvent residue signals as reference. All solvents used for reactions were purchased as anhydrous grade from Fluka. Solvents for extractions, flash chromatography and TLC were commercial grade. Mass spectra were recorded with a VG70-250 (FAB), Finnigan MAT MS 312 (EI) or Finnigan MAT LCQ (ESI) spectrometer. High resolution mass spectra were recorded with a Thermo Fisher Scientific LTQ Orbitrap XL, nanoelectrospray ion source.

Measurement of the absorption spectra

UV/Vis spectra of 10^{-5} M solution of MeNV-HaXS in DMSO was recorded on a Perkin Elmer Lambda 40 UV/Vis spectrometer using quartz standard absorption cells. The extinction coefficients were calculated for 360 nm.

The emission of the transilluminator lamps (RPR-3500 Å, RPR-3600 Å and RPR-4190 Å) was recorded with an AVA SPEC 2048 spectrometer. With the same spectrometer an absorption spectrum of the glass cover slip was recorded to verify that there is no absorbance in the irradiation wavelengths range.

General procedure for the analysis of photolysis

The irradiated solution was transferred into an HPLC microvial and injected into an Aquity H-Class UPLC system equipped with an ESI-SQD mass spectrometer. The photolysis yield was determined by integration of the Single Ion Monitoring (SIM) signal of the starting product.

Protein denaturation, cell lysis and immune-blotting

Cells were washed with ice cold PBS and lysed in a NP-40 lysis buffer [1% NP-40, 20 mM Tris-HCl pH 8.0, 138 mM NaCl, 2.7 mM KCl, 5% glycerol, 40 mM NaF, 2 mM Na_3VO_4, 20 µM Leupeptin, 18 µM Pepstatin, 5 µM Aprotinin, 1 mM PMSF, 1 mM $MgCl_2$, 1 mM $CaCl_2$, 5 mM EDTA]. Cell lysates were cleared by centrifugation at 13'000 rpm for 15 min and proteins were denatured by the addition of 5x sample buffer [312.5 mM Tris-HCl (pH 6.8), 10% SDS, 25% β-mercaptoethanol, 50% glycerol, bromphenol blue] and cooking for 6 min. Proteins were separated by SDS-PAGE and transferred to Immobilon PVDF membranes (Millipore). Mouse monoclonal antibody (mAb) to GFP (Roche Diagnostics) was used to detect GFP-fusion proteins. Secondary antibodies labeled with horseradish peroxidase (HRP-conjugated goat anti-mouse IgG) were visualized using enhanced chemiluminescence (Millipore) and a CCD camera (Fusion Fx7, Vilber).

Cloning of expression constructs

The HaloTag7 L273Y coding sequence (Promega) and SNAPf coding sequence (kind gift from K. Johnsson, Lausanne), mTFP1 (kind gift of O. Pertz, Basel), Golgi targeting sequence Giantin (kind gift from T. Inoue, Baltimore), LAMP1 (kind gift from T. Inoue, Baltimore), plasma

membrane targeting sequence CAAX (kind gift of J, Downward, London) and mTq (kind gift of J. Goedhart Amsterdam) were amplified by PCR (Phusion polymerase, Finnzymes).

For HaloTag L273Y L273Y-GFP, HaloTag7 L273Y-RFP, and SNAPf-GFP expression constructs, HaloTag7 L273Y and SNAPf were transferred to pEGFP (Clonetech), or pTag-RFP-N1 (Evrogen) expression vectors.

SNAPf-mTFP1: GFP was exchanged in a SNAPf-GFP plasmid by the mTFP1 sequence.

HaloTag7 L273Y-RFP-Giantin: the Giantin targeting sequence was introduced into the HaloTag7 L273Y-RFP expression vector.

NLS-CFP-SNAP: FKBP1x from the NLS-CFP-FKBP1x expression vector (kind gift from S. Hübner, Würzburg) was exchanged by the SNAPf sequence.

HaloTag7 L273Y-RFP-Rheb15: HaloTag7 L273Y-RFP was amplified by PCR and fused to a Rheb15 sequence to be inserted into the pTag-RFP-N1 backbone with RFP was excised.

Halo-RFP-Rheb15: HA-Raptor was replaced by HaloTag7 L273Y-RFP in a HA-Raptor-Rheb15 expression vector kindly obtained from Anna Melone.

LAMP-RFP-HaloTag7 L273Y: the LAMP1 targeting sequence was inserted into the RFP-HaloTag7 L273Y expression vector.

Mito-SYFP-SNAPf: FRB was exchanged into a Mito-SYFP-FRB expression construct (kind gift of P. Scheiffele, Basel) by the SNAPf sequence.

LifeAct-mTFP1-SNAPf: mTFP1 was fused to LifeAct by PCR, and inserted into the GFP-SNAPf expression construct (were GFP was excised).

HaloTag7 L273Y-mTq: GFP was exchanged by the mTq sequence.

Maps and expression vector sequences can be obtained from the authors upon request.

Statistical Analysis

Statistical analysis was performed with GraphPad Prism v6. For Student's t test (two sided, non-paired with Welch correction, $p < 0.05$) ≥3 independent experiments were compared.

Cell culture and transfection

HeLa cells (ATCC) were cultured in complete Dulbeccos's modified Eagle medium (DMEM) with 10% heat-inactivated fetal calf serum (HIFCS), 2 mM L-glutamine (Gln), 1% penicillin-streptomycin solution (PEST) at 37°C and 5% CO_2. Transfections were carried out with JetPEI (Brunschwig) according to manufacturer's guidelines.

Cellular heterodimerization and intracellular cleavage

HeLa cells were grown in 6-well cell culture plates (Falcon), and were transfected with expression constructs for SNAP-GFP, Halo-GFP, Halo-RFP-Giantin, SNAP-mTFP1 or NLS-CFP-SNAP. After 24 h, cells were exposed to MeNV-HaXS or HaXS8 dimerizer as described in complete cell culture medium (at 37°C). Before bulk photocleavage experiments, cells were washed twice in 1 x PBS after treatment with HaXS8 or MeNV-HaXS. Then 600 μl 1x PBS was added to each well, and the cell cells in the culture plate on ice was illuminated for 10 min with a high-intensity UV lamp (Blak-Ray B-100A high intensity UV lamp; 100 Watt, 365 nm, UVP) at a distance of 5 cm.

For Western Blot analysis, cells were lysed, and proteins were separated by SDS-PAGE. SNAP/Halo-tag dimers were detected using anti-GFP (primary) and horseradish peroxidase (HRP)-conjugated (secondary) antibodies to visualize dimer formation using enhanced chemiluminescence (Millipore) and a CCD camera (Fusion Fx7, Vilber).

For live cell microscopy, transfected and MeNV-HaXS resp. HaXS8 treated cells grown on 12 mm cover slips (Menzel) were mounted in Ludin chambers (Live Imaging Services) in the closed confirmation with complete cell culture medium without phenol red. Pictures were taken every 1 seconds before illumination of the cells and every 1 sec for Figure 3 resp. every 15 sec for Figure 4. Images were acquired on a Leica Live Imaging Microscope fitted with a HCX Plan-Fluotar 63x/1.4 oil objective and a Photometrics CCD Camera CoolSnap HQ2 with Metamorph 7.1 software (Molecular devices). Cleavage in Fig. 3a and 3b and Fig. 4a was performed with a 50 mW FRAP scanning laser (UV-Diode Laser, 355 nm, 30 Hz). Single spots within the indicated region of interest where irradiated for the indicated times. For photocleavage experiments as shown in Fig. 3d, standard DAPI excitation filters were used on a Leica EL600 microscope (equipped with a mercury metal halide lamp; filter cube: ex: 377±25 nm, em: 447±30 nm, dichrioic mirror: 409 nm; 63x objective: 63xHCX Plan-Fluotar, NA: 1.25 [oil]).

Movies were assembled and analyzed with Fiji (ImageJA, 1.44b). In Figure 3a mTFP1 fluorescence at vesicles was calculated according to the formula mTFP1 = F(mTFP1, vesicle) / F(RFP, whole cell) - F(mTFP1, cytoplasm) / F(RFP, whole cell) for every frame. In Figure 3b mTPF1

fluorescence in the cytoplasm was calculated according to the formula mTFP1 = F(mTFP1, vesicle) / F(RFP, whole cell). In Figure 4 CFP fluorescence at vesicles was calculated according to the formula CFP = F(CFP, vesicle) / F(RFP, whole cell) - F(CFP, cytoplasm) / F(RFP, whole cell) for every frame. Nuclear CFP fluorescence intensity was calculated according to the formula CFP = F(CFP, nucleus) / F(RFP, whole cell) for every image. To illustrate the fluorescence decrease from vesicles and to measure fluorescence increase in the cytoplasm or nucleus, mTFP1 or CFP fluorescence intensity was plotted over time.

3. Chemical synthesis and characterization of compounds

Synthesis of MeNV-HaXS

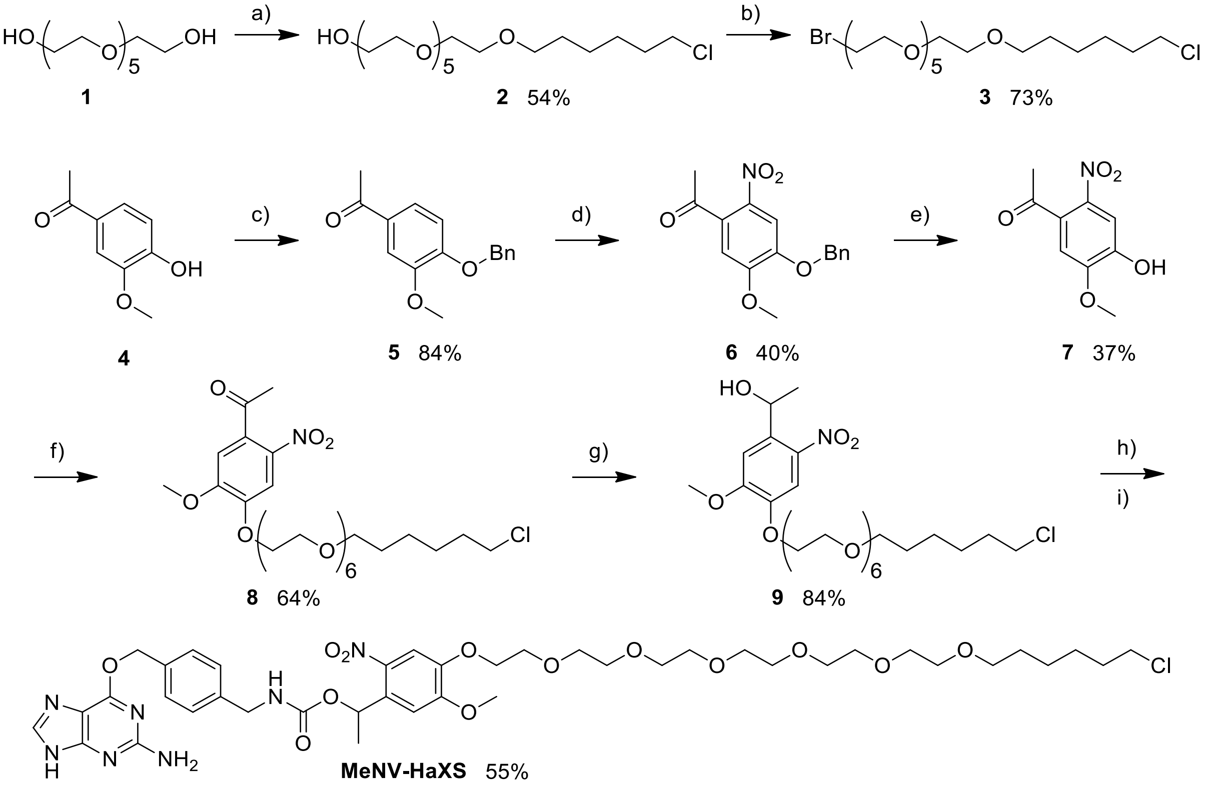

Reaction conditions: a) NaH, 6-chloro-1-iodohexane, THF/DMF, 0°C, 16 h; b) PPh_3, CBr_4, rt, 16 h; c) K_2CO_3, benzyl bromide, DMF, 80°C, 16 h; d) Acetic acid, acetic anhydride, HNO_3, rt, 16 h; e) Acetic acid, HBr, 85°C, 1.5 h; f) K_2CO_3, **3**, DMF, 60°C, 16 h; g) $NaBH_4$, MeOH/Dioxane, rt, 2 h; h) bis *p*-nitrophenyl carbonate, triethylamine, DMF, rt, 16 h; i) O6-aminomethylbenzylguanine, 60°C, 6 h.

Scheme S1. Synthetic route to MeNV-HaXS.

Preparation of 24-chloro-3,6,9,12,15,18-hexaoxatetracosan-1-ol (2)

Hexaethyleneglycol (**1**) (2.1 mL, 8.4 mmol) was dissolved in a THF/DMF mixture (3:1). Sodium hydride (60% in mineral oil, 370 mg, 9.2 mmol) was added portionwise at 0 °C. After stirring for 30 min at rt, 6-chloro-1-iodohexane (6.4 mL, 8.4 mmol) was added dropwise at 0 °C. The mixture was stirred at rt for 16 h. The excess of sodium hydride was carefully quenched with water and the crude mixture was poured into water and extracted twice with AcOEt. The combined organic layers were dried over Na_2SO_4 and concentrated under reduced pressure. The crude oil was purified by flash chromatography (CH_2Cl_2/MeOH, 20:1) to yield compound **2** (5.3 g, 54%). **^{1}H NMR** (400 MHz, CD_3OD): δ 3.66 - 3.61 (m, 18H), 3.58 – 3.54 (m, 6H), 3.47 (t, J = 6.80 Hz, 2H), 3.30 (m, 2H), 1.77 (m, 2H), 1.58 (m, 2H), 1.50-1.38 (m, 4H); **^{13}C NMR** (100.6 MHz, CD_3OD): δ 73.6, 72.1, 71.6, 71.5, 71.5,71.4, 71.1, 62.2, 45.7, 33.7, 30.5, 27.7, 26.5**.**

Preparation of 1-bromo-24-chloro-3,6,9,12,15,18-hexaoxatetracosane (3)

24-chloro-3,6,9,12,15,18-hexaoxatetracosan-1-ol **2** (950 mg, 2.37 mmol) was dissolved in THF. Triphenylphosphine (721 mg, 2.76 mmol) and carbon tetrabromide (900 mg, 2.76 mmol) were added portionwise at 0°C. The resulting mixture was stirred at rt for 16 h. The solvent is evaporated

under reduced pressure and the crude oil is purified by flash chromatography (cyclohexane/EtOAc, 3:1) to yield compound **3** (800 mg, 73%). **^{1}H NMR** (400 MHz, CD_3OD): δ 3.80 (t, J = 6.02 Hz, 2H), 3.66-3.61 (m, 18H), 3.58-3.54 (m, 4H), 3.51 (t, J = 6.04 Hz, 2H), 3.48 (t, J = 6.52), 1.77 (m, 2H), 1.58 (m, 2H), 1.36-1.52 (m, 4H); **^{13}C NMR** (100.6 MHz, CD_3OD): δ 72.3, 72.1, 71.6, 71.4, 71.2, 45.7, 33.8, 31.4, 30.6, 27.7, 26.5.

Preparation of 1-(4-(benzyloxy)-3-methoxyphenyl)ethanone (5)

Acetovanillone (**4**) (5 g, 30.1 mmol) and benzylbromide (5.15 g, 30.1 mmol) were dissolved in 50 mL DMF. K_2CO_3 (4.15 g, 30.1 mmol) was added and the mixture was stirred at 80°C for 16 h. The reaction was quenched with sat. NH_4Cl (2 L) and extracted with AcOEt three times. The combined organic layers were dried over Na_2SO_4 and concentrated under reduced pressure. The resulting product **5** (6.5 g, 84%) was used without further purification. **^{1}H NMR** (400 MHz, $CDCl_3$): δ 7.48 (d, J = 2.01 Hz, 1H), 7.43 (dd, J = 8.26 Hz, 2.01 Hz, 1H), 7.36 (d, J = 7.23 Hz, 2H), 7.31 (td, J = 7.23 Hz, 1.54 Hz, 2H), 7.25 (m, 2H), 6.82 (d, J = 8.26 Hz, 1H), 5.16 (s, 2H), 3.88 (s, 3H), 2.47 (s, 3H); **^{13}C NMR** (100.6 MHz, $CDCl_3$): δ 196.9, 152.5, 149.6, 136.4, 130.8, 128.7, 128.1, 127.2, 123.1, 112.2, 110.5, 70.8, 56.1, 26.2; **HRMS** $C_{16}H_{17}O_3$ $[M+H]^+$ calcd: 257.1172, found: 257.1166.

Preparation of 1-(4-(benzyloxy)-5-methoxy-2-nitrophenyl)ethanone (6)

Nitric acid (1.4 mL, 69%) was added dropwise to a solution of *o*-benzylacetovanillone (**5**) (1.5 g, 5.8 mmol) in acetic acid (4.9 mL) and acetic anhydride (1.4 mL) at 0°C. The mixture was stirred at rt overnight. The reaction was poured into ice water and extracted with AcOEt three times. The combined organic layers were washed with saturated $NaHCO_3$, brine and dried over Na_2SO_4. The solvent was evaporated under reduced pressure and the crude product was purified by flash chromatography (cyclohexane/EtOAc 4:1) to yield the desired product **6** (718 mg, 40%). **^{1}H NMR** (400 MHz, $CDCl_3$) : δ 7.67 (s, 1H), 7.46-7.29- (m, 5H), 6.77 (s, 1H), 5.22 (s, 2H), 3.98 (s, 3H), 2.49 (s, 3H); **^{13}C NMR** (100.6 MHz, $CDCl_3$): δ 200.1, 154.6, 148.6, 138.3, 135.3, 133.1, 128.9, 128.6, 127.6, 108.8, 71.4, 56.7, 30.4; **HRMS** $C_{16}H_{16}O_5N$ $[M+H]^+$ calcd: 302.1023, found: 302.1017.

Preparation of 1-(4-hydroxy-5-methoxy-2-nitrophenyl)ethanone (7)

6-Nitro-*o*-benzylacetovanillone (**6**) (4.3 g, 14.3 mmol) was dissolved in acetic acid (30 mL, 99%), and heated to 85°C. HBr (15 mL, 48%) was added to the mixture and stirred for 1.5 h. The mixture was poured into ice water and extracted with AcOEt three times. The combined organic layers were washed with saturated $NaHCO_3$, brine and dried over Na_2SO_4. The solvent was evaporated under reduced pressure. The crude product was purified by flash chromatography (cyclohexane/EtOAc, 2:1) to yield the desired compound **7** (1.1 g, 37%). **^{1}H NMR** (400 MHz, $CDCl_3$): δ 9.76 (s, 1H), 7.60 (s, 1H), 6.81 (s, 1H), 3.98 (s, 3H), 2.48 (s, 3H); **^{13}C NMR** (100.6 MHz, $CDCl_3$): δ 199.7, 152.3, 147.8, 138.8, 130.4, 110.8, 108.8, 56.2, 29.9; **HRMS** $C_9H_{10}O_5N$ $[M+H]^+$ calcd: 212.0553, found: 212.0553.

Preparation of 1-(4-((24-chloro-3,6,9,12,15,18-hexaoxatetracosyl)oxy)-5-methoxy-2-nitrophenyl)ethanone (8)

o-Hydroxy-6-nitrovanillone (**7**) (455 mg, 2.2 mmol) and K_2CO_3 (297 mg, 2.2 mmol) were added to a solution of **3** (1.0 g, 2.2 mmol) in DMF (6 mL). The mixture was stirred at 60°C overnight. The reaction mixture was poured into a solution of sat. NH_4Cl (150 mL) and extracted with EtOAc three times and dried over Na_2SO_4. The solvent was evaporated under reduced pressure and the crude product was purified by flash chromatography (cyclohexane/EtOAc, 1:3) to yield the desired product **8** (830 mg, 64%). **^{1}H NMR** (400 MHz, DMSO-d_6): δ 7.69 (s, 1H), 6.76 (s, 1H), 4.28 (m, 2H), 3.96 (s, 3H), 3.92 (m, 2H), 3.74 (m, 2H), 3.69 – 3.62 (m, 18H), 3.56 (m, 2H), 3.53 (t, J = 6.8 Hz, 2H), 3.45 (t, J = 6.8 Hz, 2H), 2.50 (s, 3H), 1.77 (m, 2H), 1.59 (m, 2H), 1.49 – 1.34 (m, 4H); **^{13}C NMR** (100.6 MHz, DMSO-d_6): δ 200.1, 154.4, 149.0, 138.3, 133.0, 108.9, 108.7, 71.2, 71.0, 70.7, 70.6, 70.1, 69.4, 69.3, 56.7, 53.6, 45.1, 32.6, 30.4, 29.5, 26.7, 25.5; **HRMS** $C_{27}H_{45}ClNO_{11}$ $[M+H]^+$ calcd: 594.2676, found: 594.2664.

Preparation of 1-(4-((24-chloro-3,6,9,12,15,18-hexaoxatetracosyl)oxy)-5-methoxy-2-nitrophenyl)ethanol (9)

Compound **8** (675 mg, 1.1 mmol) was dissolved in MeOH/Dioxane mixture (7 mL / 7 mL). $NaBH_4$ (51 mg, 2.0 mmol) was added portionwise at 0°C. The mixture was stirred at rt for 2 h. Then, the mixture was poured into water, neutralized with a 1 M solution of HCl and extracted with CH_2Cl_2 three times. The combined organic layers were dried over Na_2SO_4 and the solvent was evaporated under reduced pressure. The crude product was purified by flash chromatography (CH_2Cl_2/MeOH, 20:1) to yield compound **9** (568 mg, 84%). **^{1}H NMR** (400 MHz, DMSO-d_6): δ 7.61 (s, 1H), 7.29 (s, 1H), 5.53 (q, J = 6.3 Hz, 1H), 4.21 (m, 2H), 3.95 (s, 3H), 3.88 (m, 2H), 3.70 (m, 2H), 3.59 – 3.66 (m, 18H), 3.55 (m, 2H), 3.51 (m, 2H), 3.43 (m, 2H), 2.50 (s, 3H), 1.75 (m, 2H), 1.57 (m, 2H), 1.52 (d, J = 6.3 Hz, 3H), 1.32 – 1.46 (m, 4H); **^{13}C NMR** (100.6 MHz, DMSO-d_6): δ 153.4, 146.3, 138.9, 138.1, 109.1, 108.6, 70.2, 70.0, 69.8, 69.5, 68.8, 68.4, 66.4, 56.0, 54.9, 45.3, 32.0, 29.1, 26.1, 25.2, 25.0; **HRMS** $C_{27}H_{47}ClNO_{11}Na$ $[M+Na]^+$ calcd: 619.2730, found: 619.2724.

Preparation of 1-(4-((24-chloro-3,6,9,12,15,18-hexaoxatetracosyl)oxy)-5-methoxy-2-nitrophenyl)ethyl (4-(((2-amino-9H-purin-6-yl)oxy)methyl)benzyl)carbamate (MeNV-HaXS)

Compound **9** (829 mg, 1.4 mmol) was added slowly to a solution of bis *p*-nitrophenyl carbonate (423 mg, 1.4 mmol) in 10 mL of DMF at 0°C. Triethylamine (193 μl, 1.4 mmol) was added and the mixture was stirred at rt overnight. O6-aminomethylbenzylguanine (379 mg, 1.4 mmol) was added portionwise. The solution was stirred at 70°C for 6 h. The reaction was quenched with H_2O and extracted with EtOAc three times. The combined organic layers were dried over Na_2SO_4 and the solvent was evaporated under reduced pressure. The crude product was purified by flash chromatography (CH_2Cl_2/MeOH, 20:1) to yield **MeNV-HaXS** (687 mg, 55%). **^{1}H NMR** (400 MHz, DMSO-d_6): δ 7.66 (s, 1H), 7.43 (d, J = 7.9, 2H), 7.21 (d, J = 7.9, 2H), 7.14 (s, 1H), 6.28 (q, J = 6.5 Hz, 1H), 5.51 (m, 2H), 4.14 – 4.31 (m, 5H), 3.80 – 3.86 (m, 6H), 3.68 – 3.70 (m, 2H), 3.50 – 3.64 (m, 24H), 3.43 (t, J = 6.6 Hz, 2H), 1.69 – 1.74 (m, 2H), 1.59 (d, J = 6.5 Hz, 3H), 1.52 – 1.59 (m, 2H), 1.30 – 1.46 (m, 4H); **^{13}C NMR** (100.6 MHz, DMSO-d_6): δ 148.7, 141.0, 140.4, 129.7, 128.4, 110.8, 109.5, 72.2, 71.8, 71.6, 71.5, 71.2, 70.7, 70.3, 70.1, 68.8, 56.9, 45.8, 45.1, 33.8, 30.6, 27.7, 26.5, 22.4; **HRMS** $C_{41}H_{59}ClN_7O_{13}$ $[M+H]^+$ calcd: 892.3854, found: 892.3842.

4. Supplementary experiments and data

Determination of the quantum yield of MeNV-HaXS at 360 nm:

The quantum yield (ϕ) of MeNV-HaXS was determined by dividing the reaction rate of the photolysis (r) by the number of photons entering the photoreactor per unit of time (quantum flow).

$$\phi = \frac{\text{reaction rate}}{\text{quantum flow}} \qquad (1)$$

Determination of the quantum flow of the Lumos 43A photoreactor:

Ferrioxalate actinometry was used to determine the quantum flow of the Lumos 43A photoreactor (LED 360 nm lamp). To this end, potassium ferrioxalate (147.5 mg, 0.34 mmol) was dissolved in 40 mL water. 40 mL of 1 N sulfuric acid were added and the solution was further diluted to 50 mL with water. 3 mL of this solution was pipetted into the absorption cell and irradiated for a given time. 2 mL of the irradiated solution was mixed with 2 mL of a 5.6 mM aqueous *o*-phenanthroline solution and 1 mL of a sodium acetate buffer. As reference 2 mL of non-irradiated ferrioxalate solution was treated with 2 mL of a 5.6 mM aqueous *o*-phenanthroline solution and 1 mL of a sodium acetate buffer. The 2 solutions were stored for 1 hour in absolute darkness prior to measure the absorbance of the solutions at 510 nm (Figure S1 A). The quantum flow was calculated using equation (2).

$$\text{Quantum flow [Einstein/s]} = \frac{A \cdot V_1 \cdot V_3}{t \cdot \phi \cdot \varepsilon \cdot l \cdot V_2} \qquad (2)$$

A = absorbance of the irradiated solution at 510 nm
t = irradiation time in seconds
ϕ = quantum yield at the irradiation wavelength (1.26 at 360 nm)
ε = extinction coefficient of the complex at 510 nm ($1.11 \cdot 10^4$ L/Mol/cm)

V_1 = irradiated volume (3 mL)
V_2 = used volume of V_1 (2 mL)
V_3 = end volume (20 mL)
l = thickness of the cell (1 cm)

The dose of photon emitted by the lamp was determined and plotted against time (Figure S1 B). The slope of the regression line, corresponding to the quantum flow of the Lumos 43A photoreactor is $1.33 \cdot 10^{-8}$ E/s or $4.79 \cdot 10^{-5}$ E/h.

Determination of the photolysis rate of MeNV-HaXS:

3 mL of a 0.5 M of a MeNV-HaXS in a DMSO/water (1:10) solution were transferred into a standard absorbance cell and irradiated in the Lumos 43A photoreactor at 360 nm for 1, 2, 3.5 and 5 minutes. The conversion was determined by UPLC-MS by integration of the absorption peak of **MeNV-HaXS**. Disappearance of the **MeNV-HaXS** was plotted as a function of the time (Figure S1 C). The slope of the regression line corresponds to the rate constant (k). The reaction rate was calculated using equation (3). The reaction rate was quantified as $6 \cdot 10^{-8}$ mol/min.

$$r = k[A] \qquad (3)$$

The reaction rate was quantified as $6 \cdot 10^{-8}$ mol/min. Using equation (1) with the reaction rate $6 \cdot 10^{-8}$ mol/min and the quantum flow of $1.33 \cdot 10^{-8}$ E/s, the photolysis quantum yield of MeNV-HaXS was calculated to be 0.075.

Determination of the molar absorption coefficient of MeNV-HaXS at 360 nm

A 10^{-5} M solution of MeNV-HaXS in DMSO was recorded on a Perkin Elmer Lambda 40 UV/Vis spectrometer using quartz standard absorption cells (l = 1cm) (Figure S1 D). The extinction coefficient at 360 nm was calculated using the Beer-Lambert law (4).

$$A = \varepsilon . l . c \qquad (4)$$

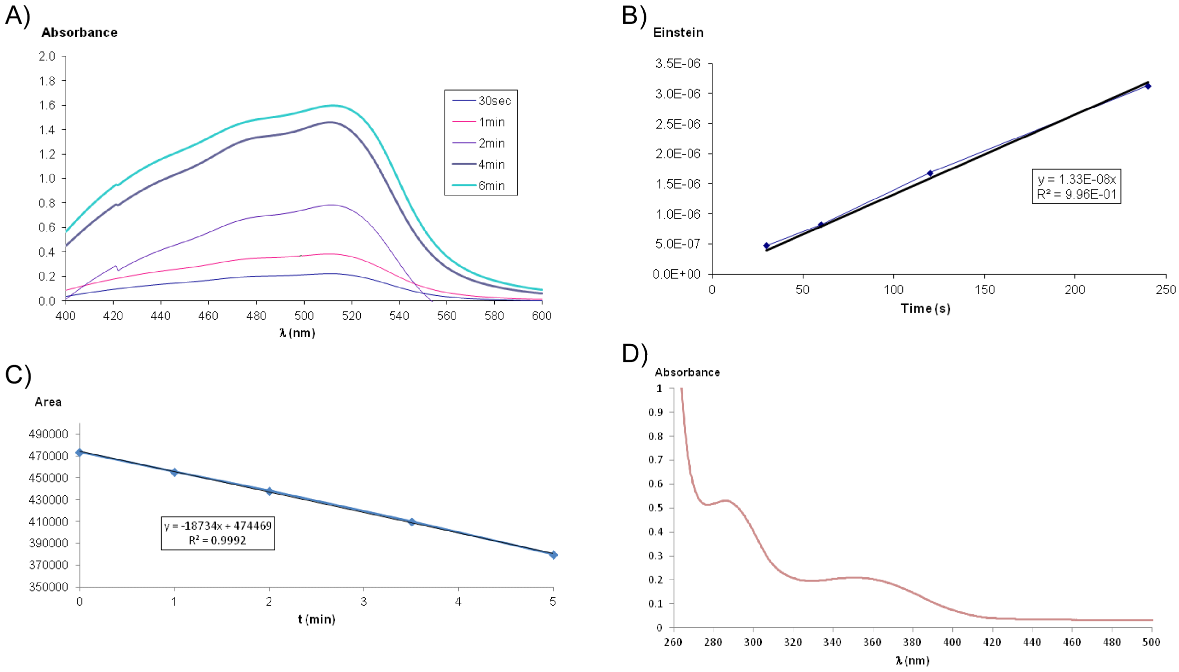

Figure S1: A) UV/Vis spectrum of ferrous *o*-phenanthroline complex formed after irradiation of Potassium ferrioxalate B) Dose of photons emitted by the lamp plotted against time to determine the quantum flow (E/s) of the Lumos 43A photoreactor (LED 360 nm lamp) C) Amount of MeNV-HaXS as determined with UPLC-MS plotted against time to determine the photolysis rate of MeNV-HaXS D) UV/Vis spectrum of MeNV-HaXS.

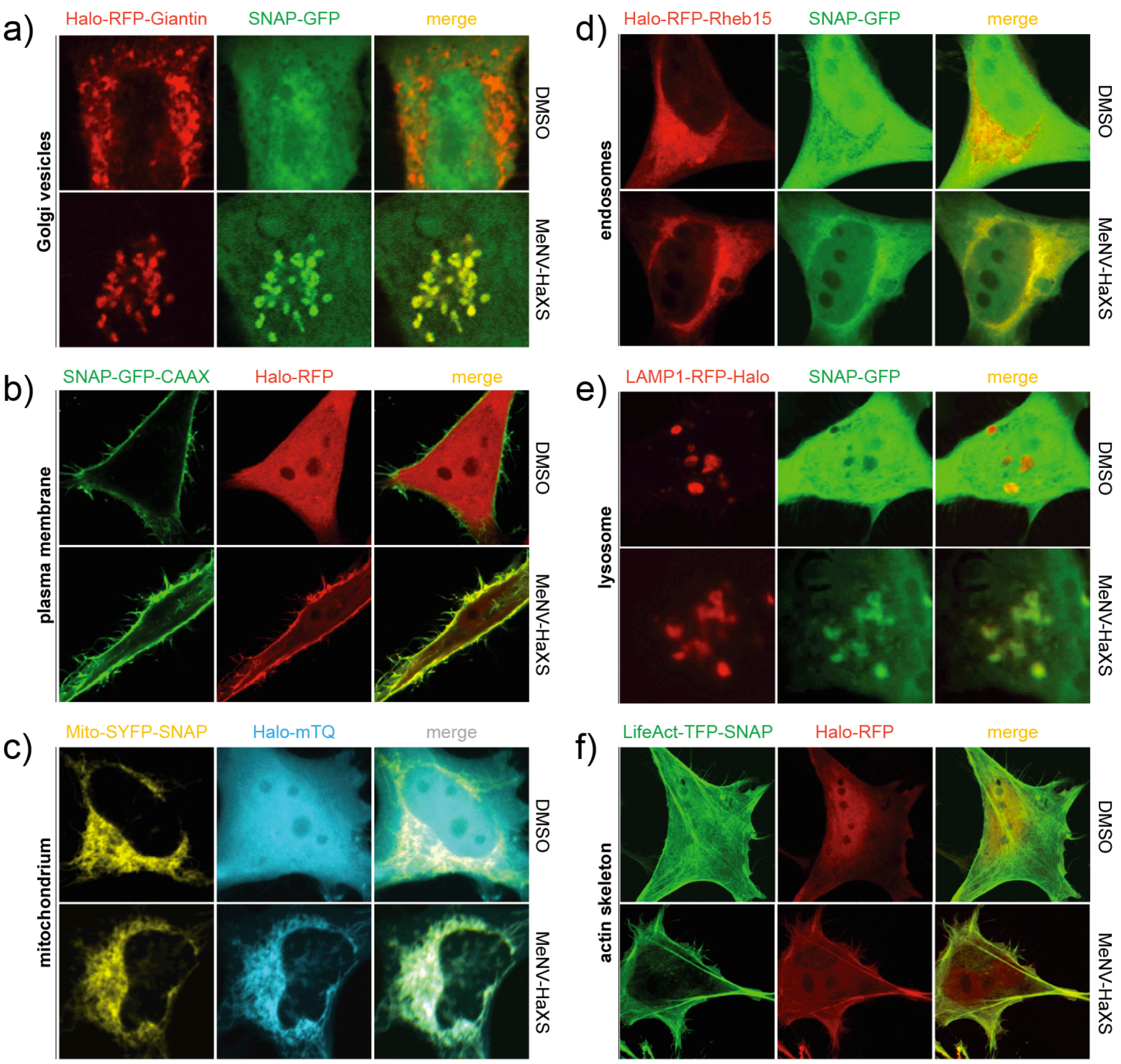

Figure S2: Cytosolic protein translocation to different cellular compartments: a) Giantin was used to target the Halo-RFP-Giantin to the Golgi, b) the CAAX box of K-Ras targeted SNAP-GFP-CAAX to the plasma membrane, c) Mito-SYFP-SNAP localized on mitochondria, d) the Rheb15-tagged Halo-RFP-Rheb15 was localized on early and late endosomes, e) LAMP1 was on lysosomes, and f) LifeAct served as an anchor on the F-actin cytoskeleton. HeLa cells expressing the indicated organelle anchors and the indicated cytosolic cargo proteins were grown on 12 mm coverslips (Menzel), before they were incubated with MeNV-HAXS (37°C, 15 min), washed twice with PBS, and fixed with 4% p-formaldehyde (PFA, in PBS), and mounted in Mowiol (Plüss-Stauffer) containing 1% propyl gallate (Sigma-Aldrich). Translocation of cytosolic Halo-RFP, Halo-mTq or Halo-RFP fusion proteins (cargo) to the respective anchors is documented.

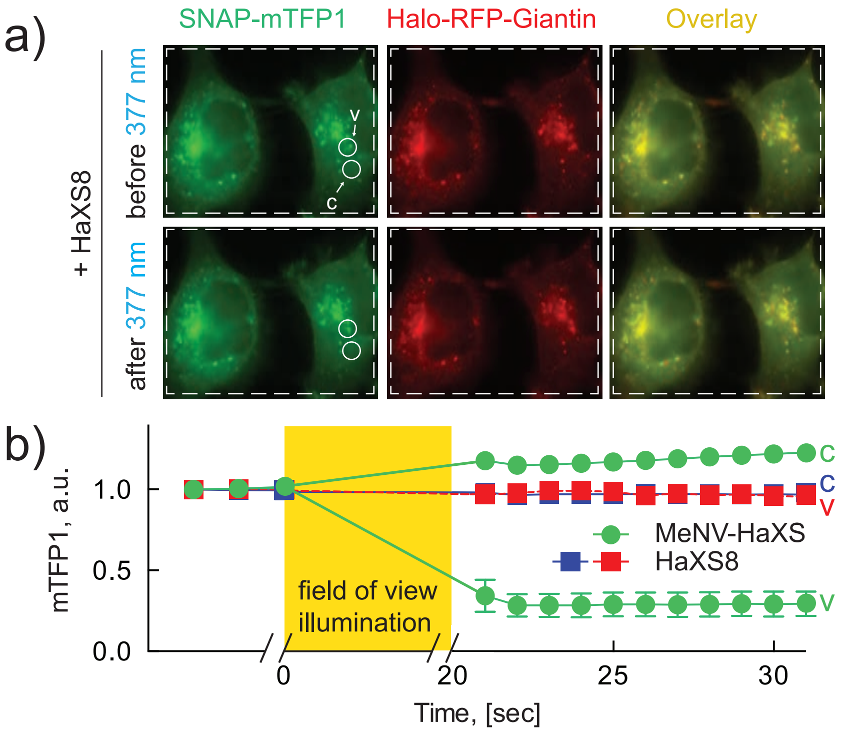

Figure S3: Release of Golgi-trapped SNAP-mTFP1 protein after UV illumination. a) At t = –15 min, HeLa cells expressing SNAP-mTFP1 and Halo-RFP-Giantin were exposed to 5 µM HaXS8 (5 µM MeNV-HaXS see Figure 3, main part), which caused the translocation of cytosolic SNAP-mTFP1 to the Golgi (labeled as “before 377 nm”). SNAP-mTFP1 intensity was monitored within the indicated circular regions of interest by live cell microscopy. At t = 0 illumination with UV light using a standard DAPI filter set on a conventional fluorescence microscope (t = 20 sec, 377 ± 25 nm) was initiated for 20 s, and cells are shown after illumination (after 377 nm). b) Quantification of mTFP1 fluorescence intensity of selected regions of interest (circles) at Golgi-derived vesicles (v) and in cytoplasm (c) before and after illumination as described in a) are shown (values represent means ± SEM, n = 10, error bars removed where smaller than symbols used). Curves obtained with MeNV-HaXS (see Figure 3) are shown in green.

Legends to movie files

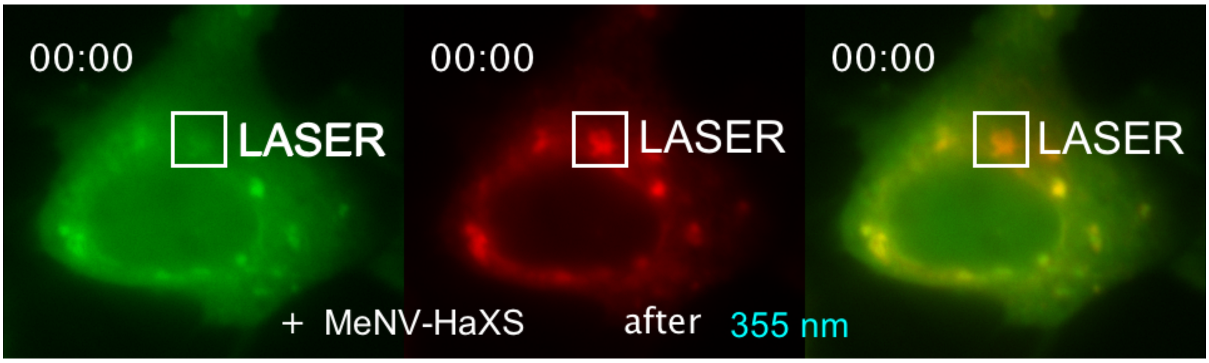

Movie S1: HeLa cells expressing SNAP-mTFP1 and Halo-RFP-Giantin (Golgi anchor) were exposed to 5 µM MeNV-HaXS (t = -15:00 min). MeNV-HaXS induced translocation of cytosolic SNAP-mTFP1 to the Golgi as imaged by live cell microscopy is shown in Movie S1. UV illumination at t = 00:00 of a subset of vesicles with a scanning FRAP laser (area marked by white square, LASER) released anchored SNAP-mTFP1 into the cytosol.

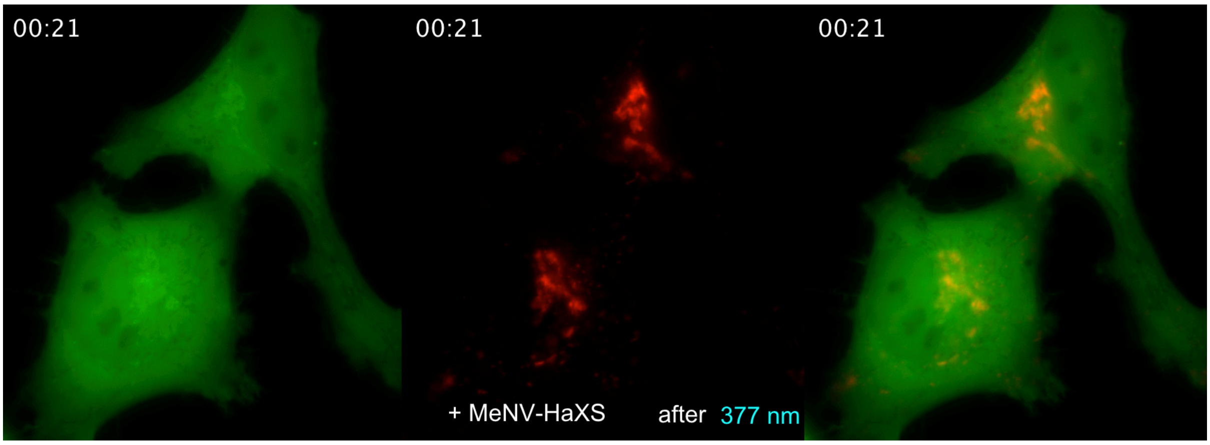

Movie S2: HeLa cells expressing SNAP-mTFP1 and Halo-RFP-Giantin were exposed to 5 µM MeNV-HaXS. Illumination of cells starting at t = 00:00 (for 20s, DAPI excitation filter, see main text) released Golgi-anchored SNAP-mTFP1.

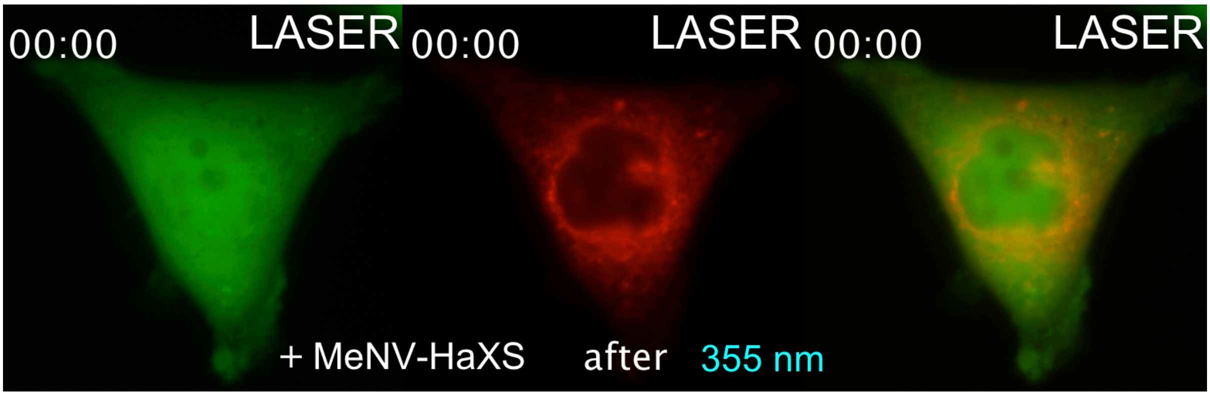

Movie S3: HeLa cells expressing the nuclear NLS-CFP-SNAP probe and Halo-RFP-Giantin were exposed to 5 µM MeNV-HaXS. The MeNV-HaXS induced translocation of nuclear NLS-CFP-SNAP to the Golgi was monitored by live cell microscopy. Illumination of the cell body was initiated at t = 00:00 (150 areas * 5 ms at 355 nm) using a scanning FRAP laser, leading to the immediate release of NLS-CFP-SNAP from Golgi vesicles into the cytosol. The reimport into the nucleus displays slower kinetics due to processes controlled by the nuclear import machinery.

5. Spectra of intermediate compounds and MeNV-HaXS

5a. ^{1}H NMR and ^{13}C NMR spectra

Figure S4: ^{1}H NMR spectrum of **2** (400 MHz, CD_3OD)

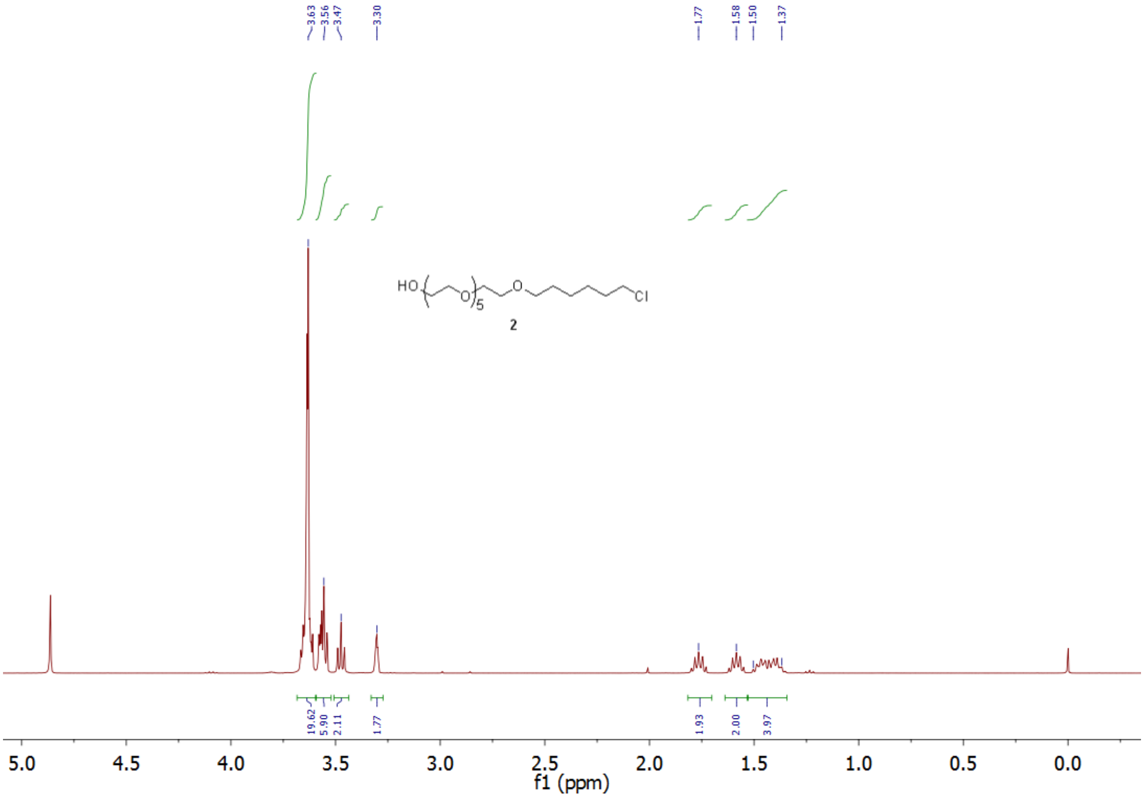

Figure S5: ^{13}C NMR spectrum of **2** (100.6 MHz, CD_3OD)

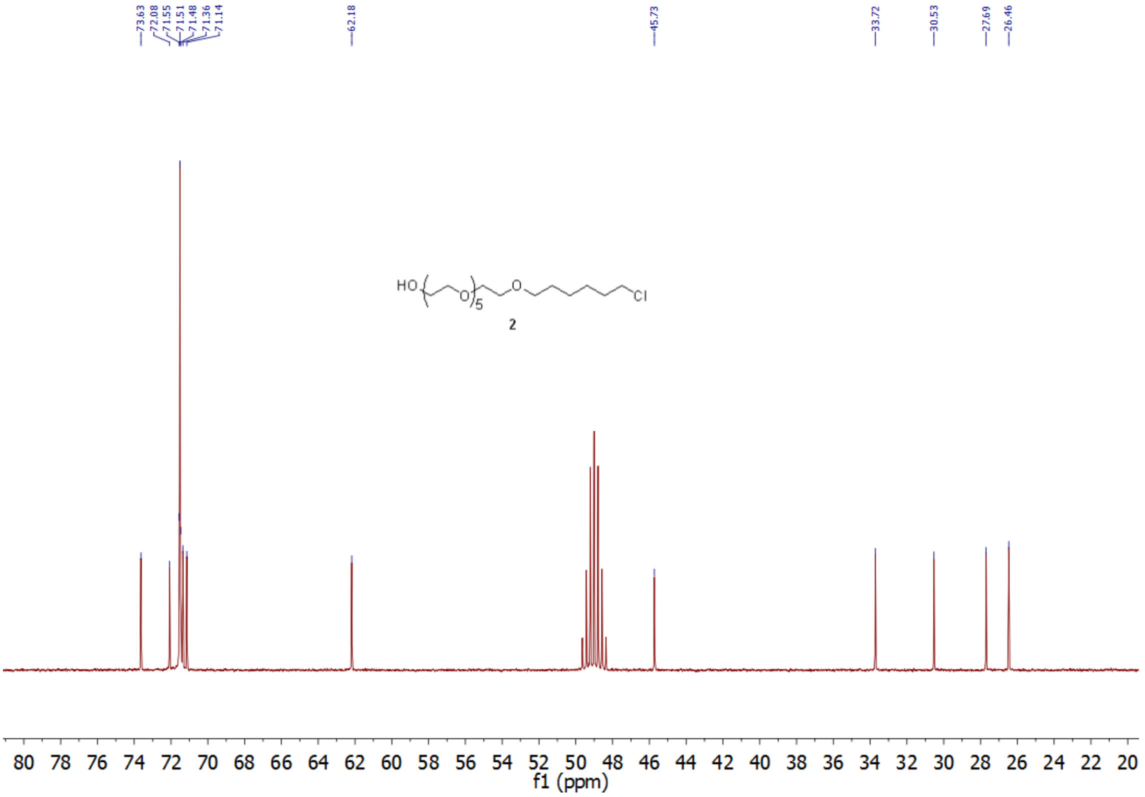

Figure S6: ^{1}H NMR spectrum of **3** (400 MHz, CD_3OD)

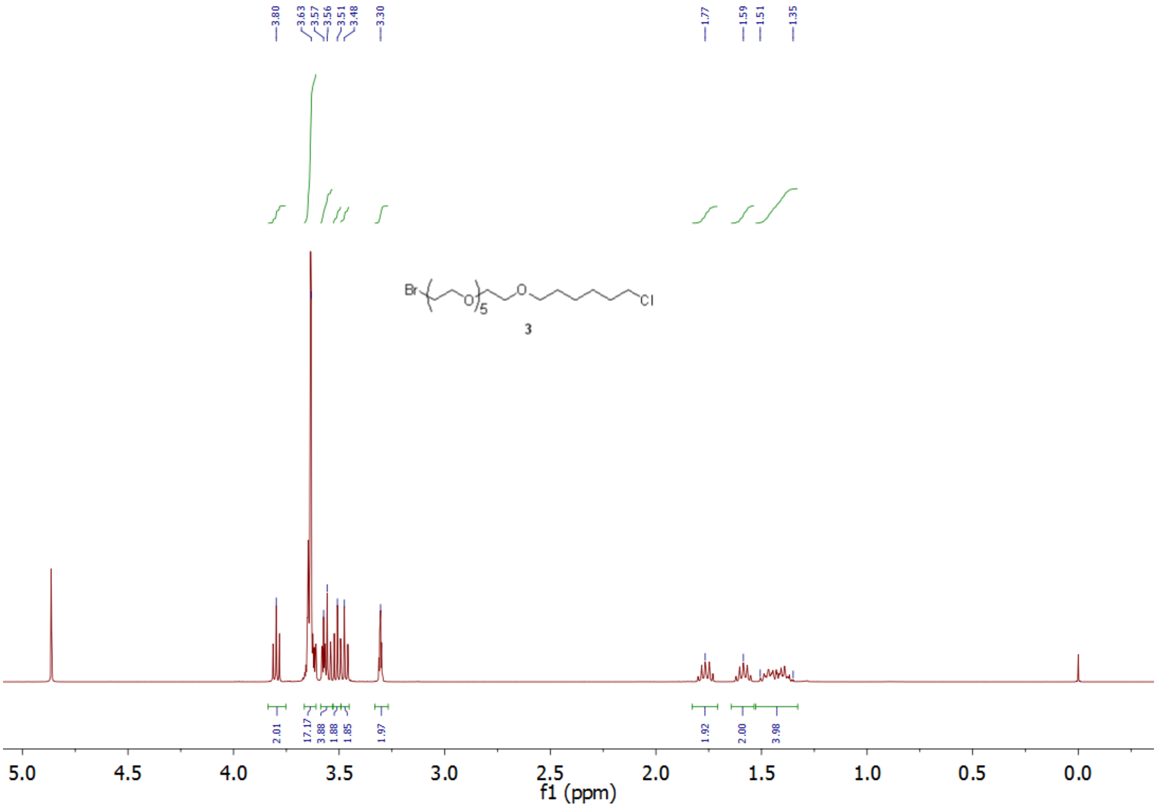

Figure S7: 13C NMR spectrum of **3** (100.6 MHz, CD3OD)

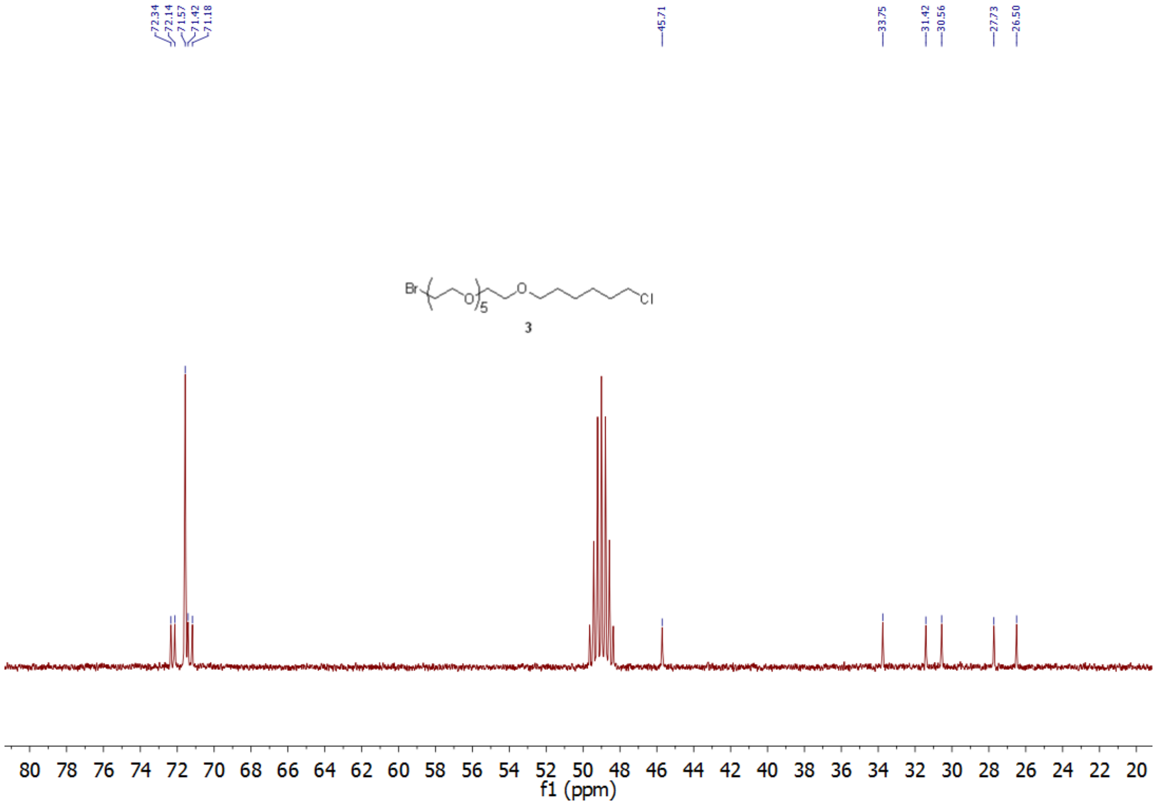

Figure S8: ^{1}H NMR spectrum of **5** (400 MHz, $CDCl_3$)

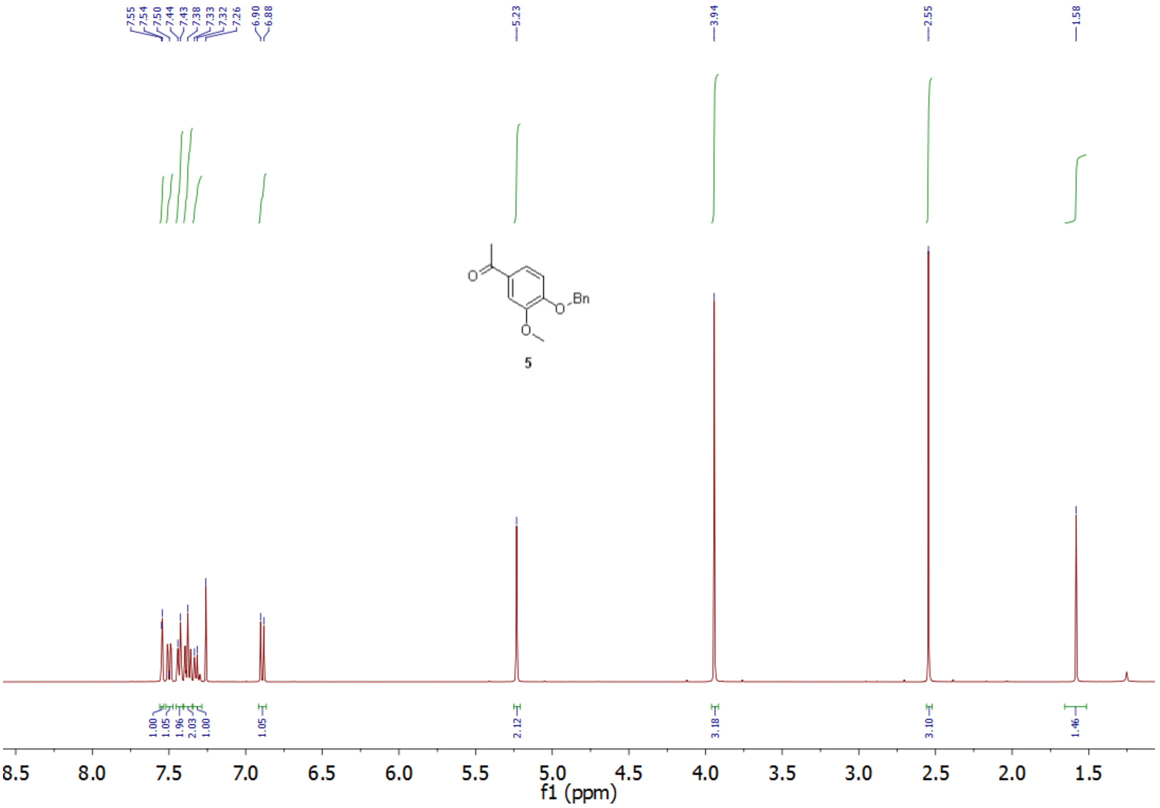

Figure S9: 13C NMR spectrum of **5** (100.6 MHz, CDCl3)

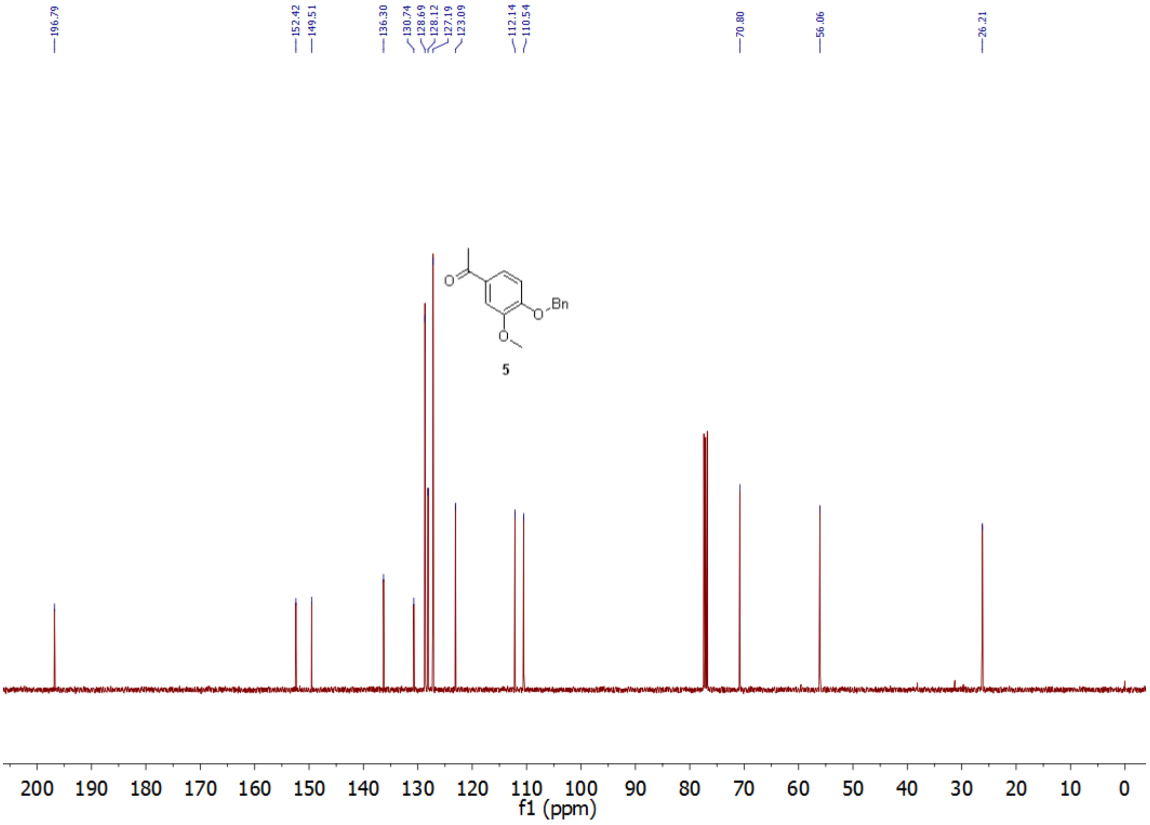

Figure S10: ^{1}H NMR spectrum of **6** (400 MHz, $CDCl_3$)

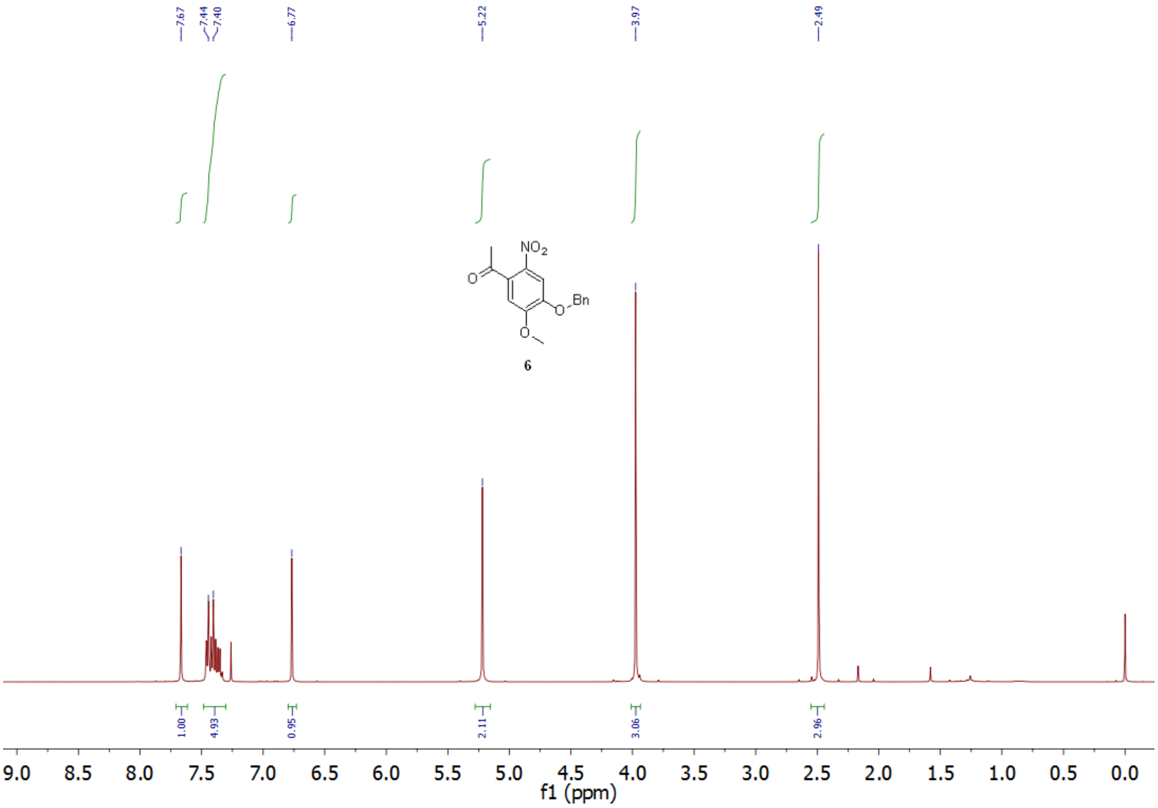

Figure S11: ^{1}H NMR spectrum of **6** (100.6 MHz, $CDCl_3$)

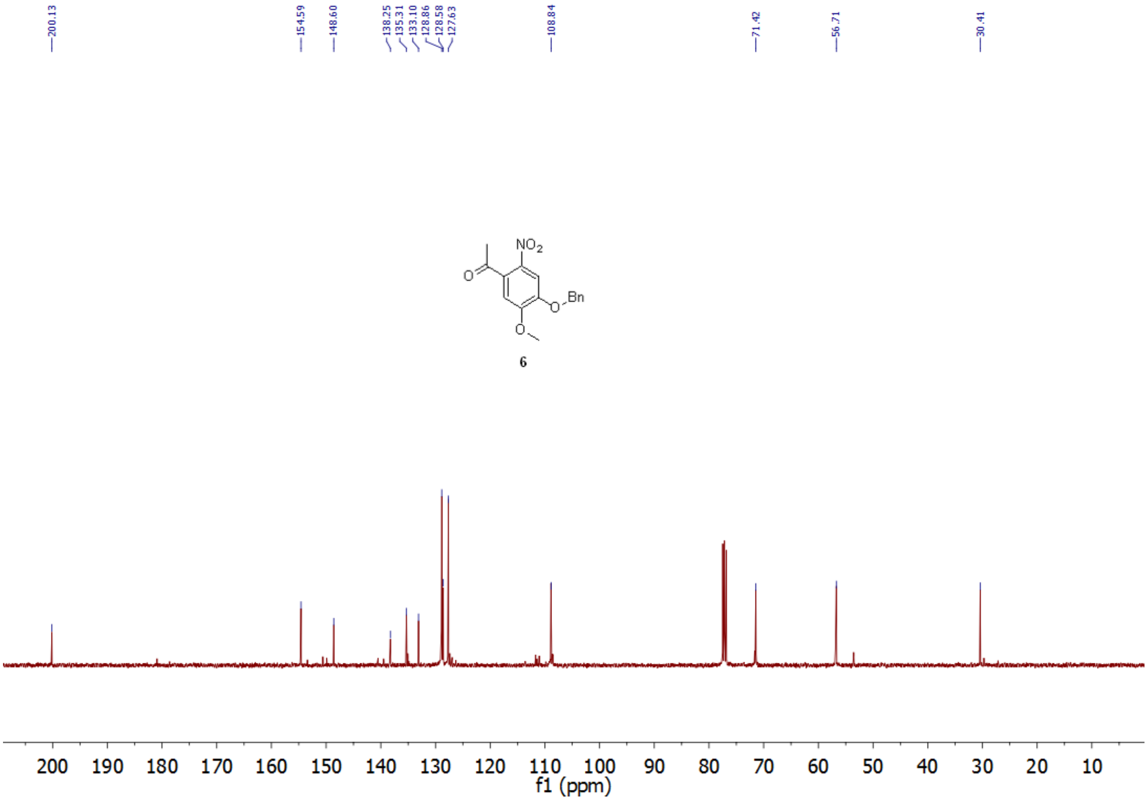

Figure S12: ^{1}H NMR spectrum of **7** (400 MHz, $CDCl_3$)

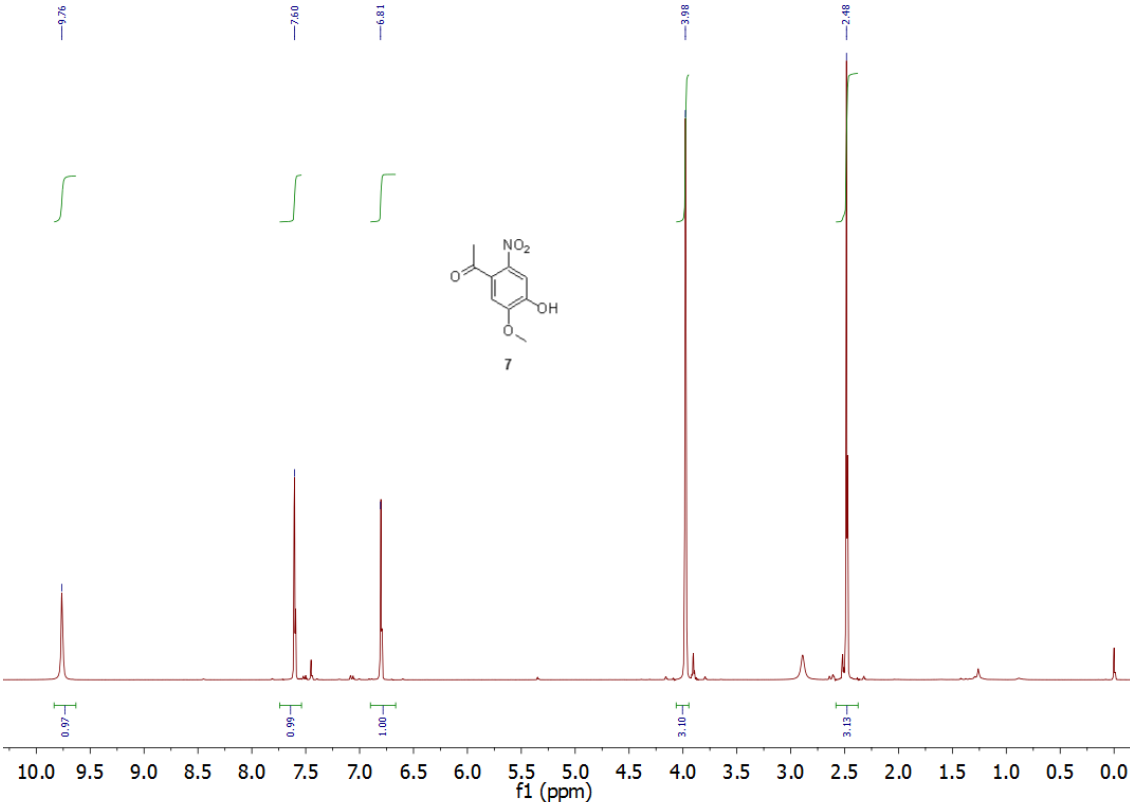

Figure S13: ^{13}C NMR spectrum of **7** (100.6 MHz, $CDCl_3$)

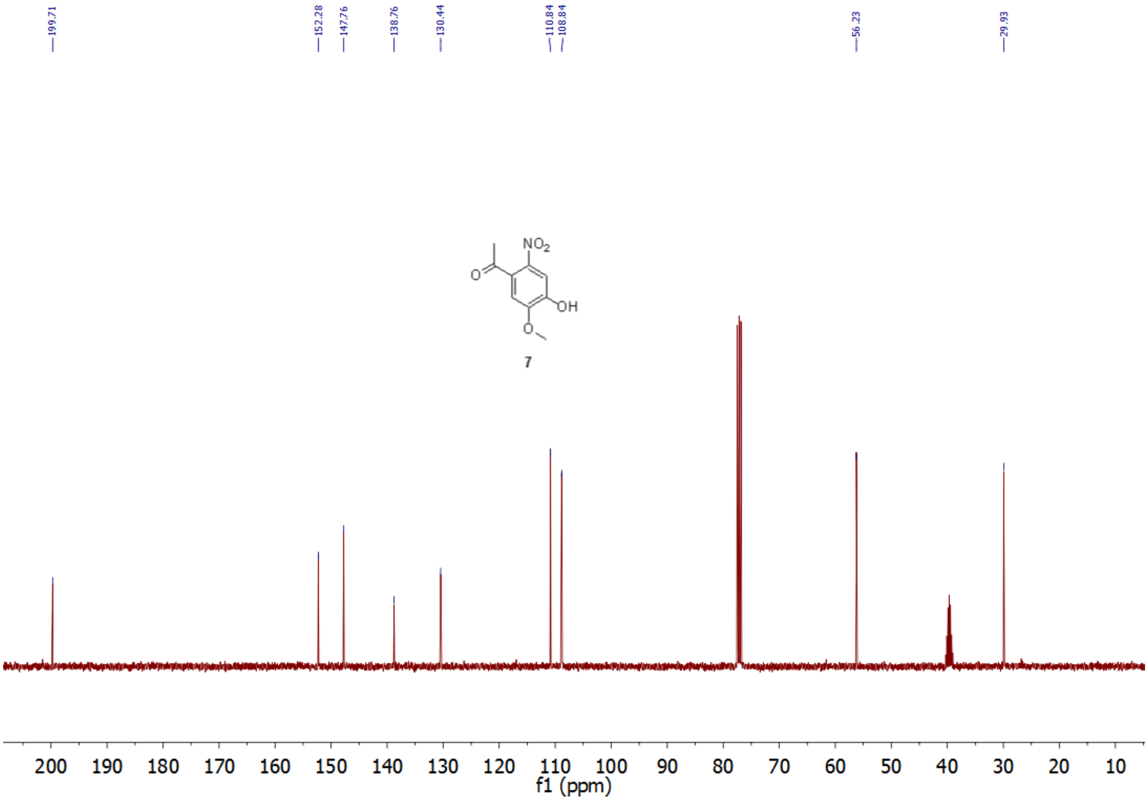

Figure S14: ^{1}H NMR spectrum of **8** (400 MHz, DMSO-d_6)

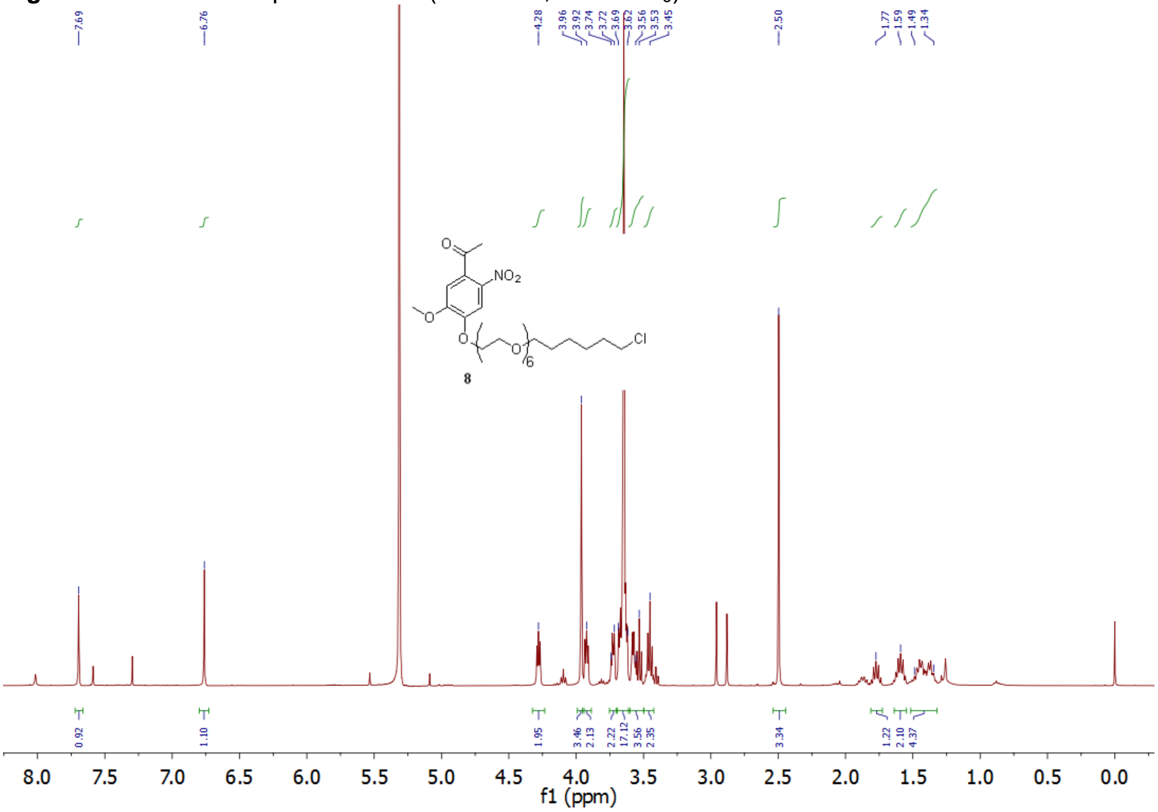

Figure S15: ^{13}C NMR spectrum of **8** (100.6 MHz, DMSO-d_6)

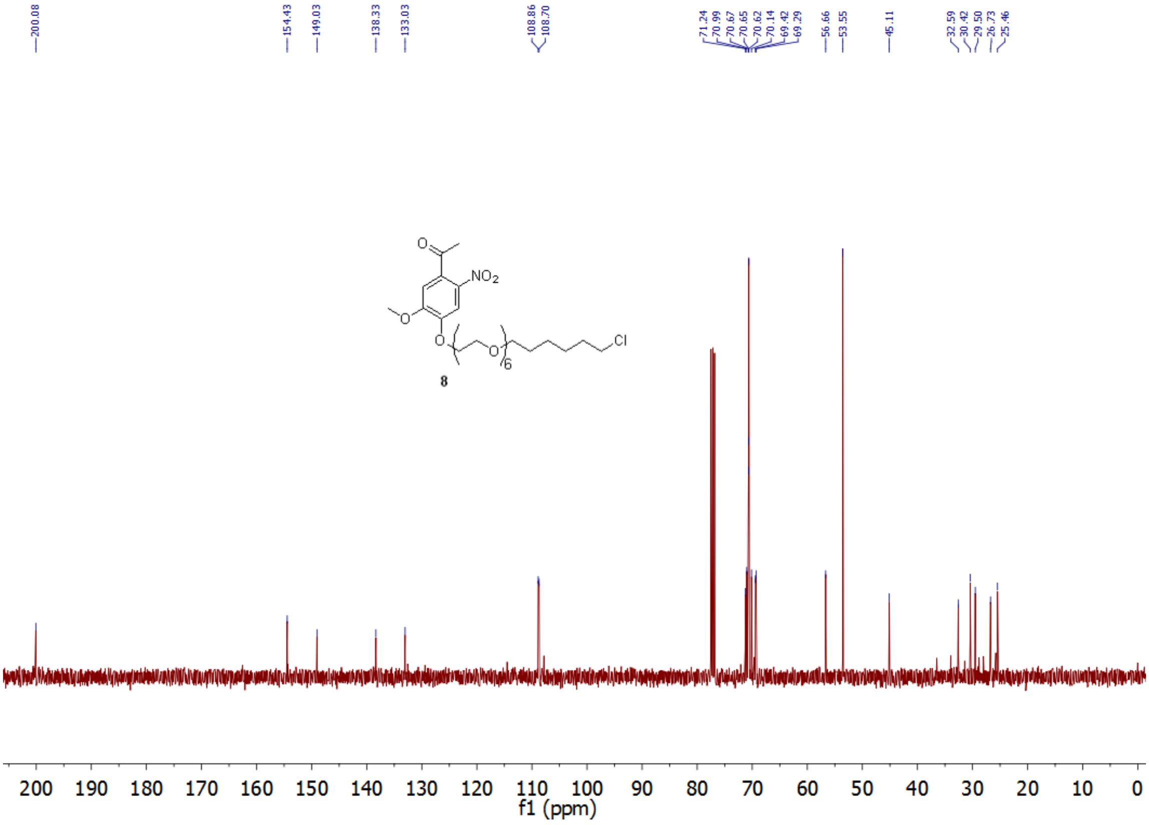

Figure S16: ^{1}H NMR spectrum of **9** (400 MHz, DMSO-d$_6$)

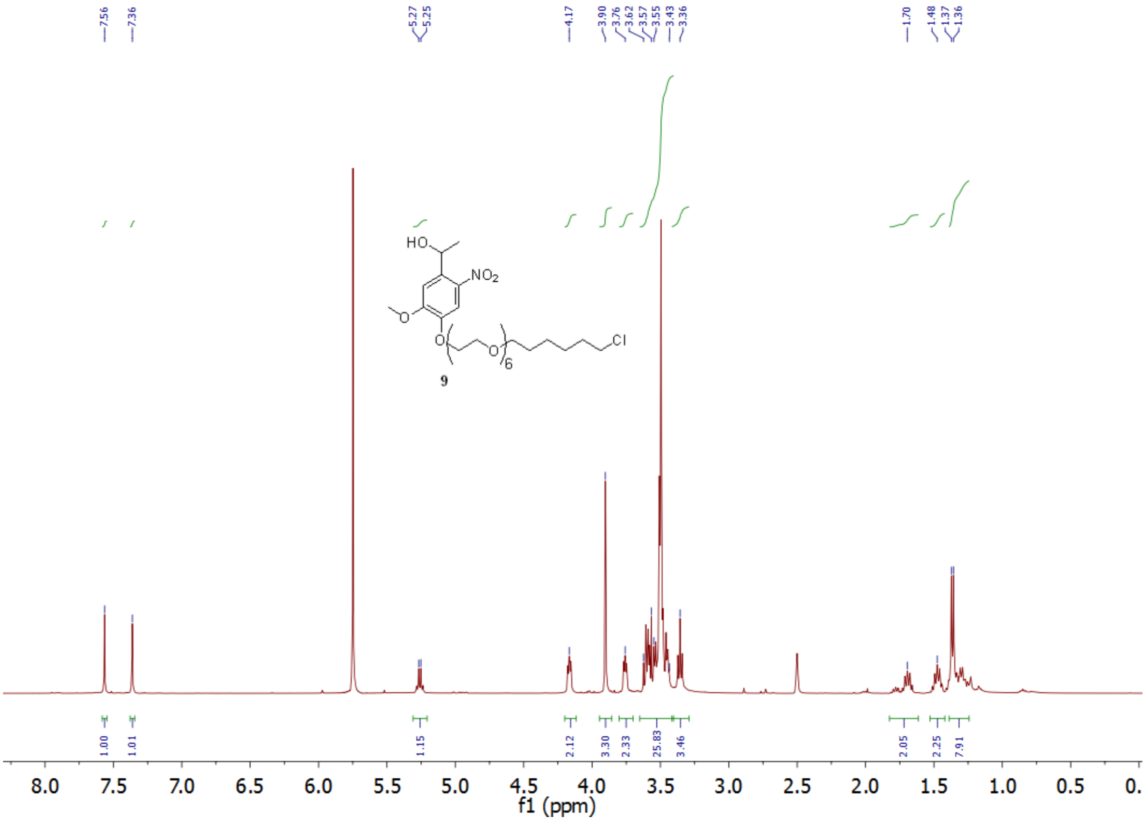

Figure S17: ^{13}C NMR spectrum of **9** (400 MHz, DMSO-d$_6$)

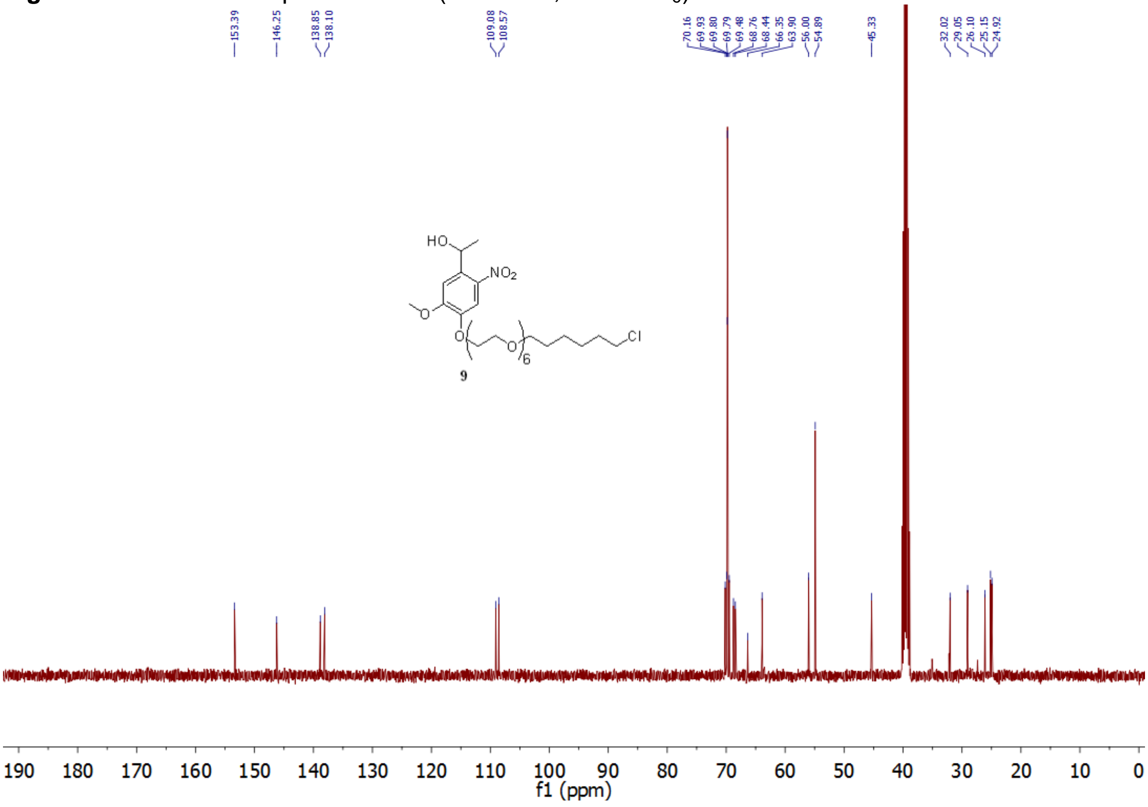

Figure S18: ^{1}H NMR spectrum of **MeNV-HaXS** (400 MHz, DMSO-d_6)

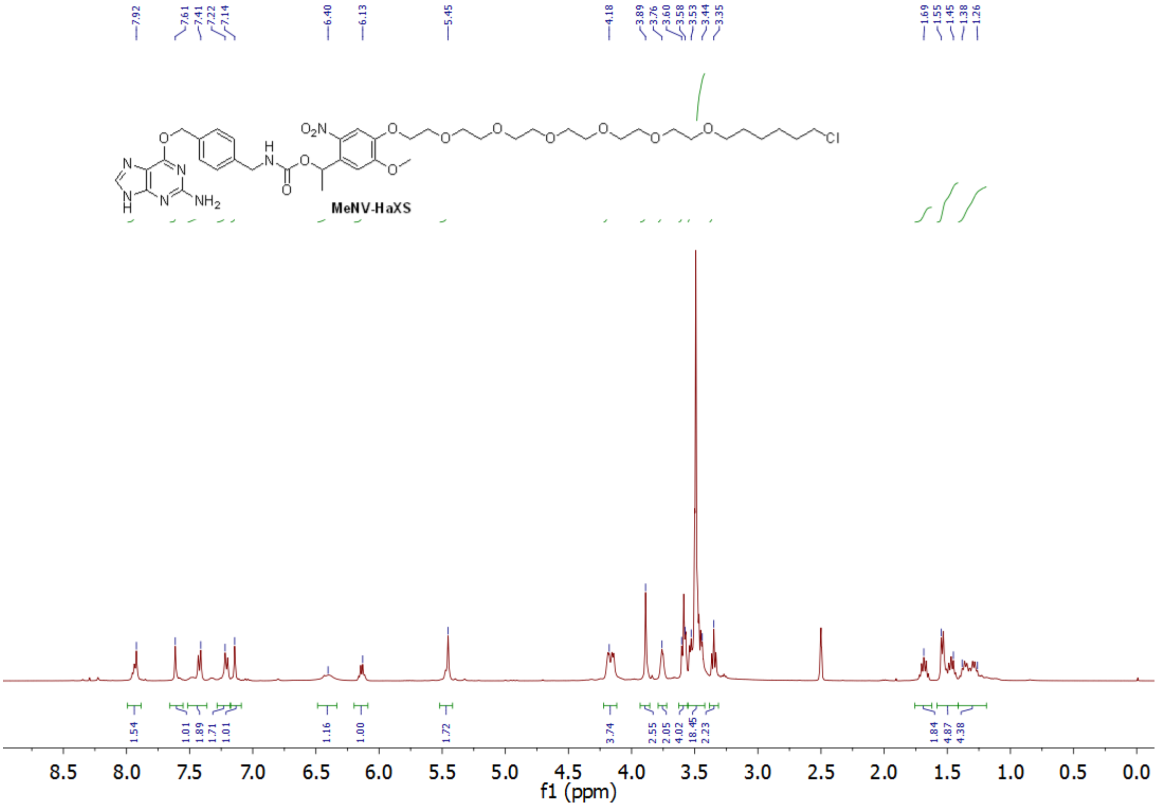

Figure S19: ^{13}C NMR spectrum of **MeNV-HaXS** (100.6 MHz, DMSO-d_6)

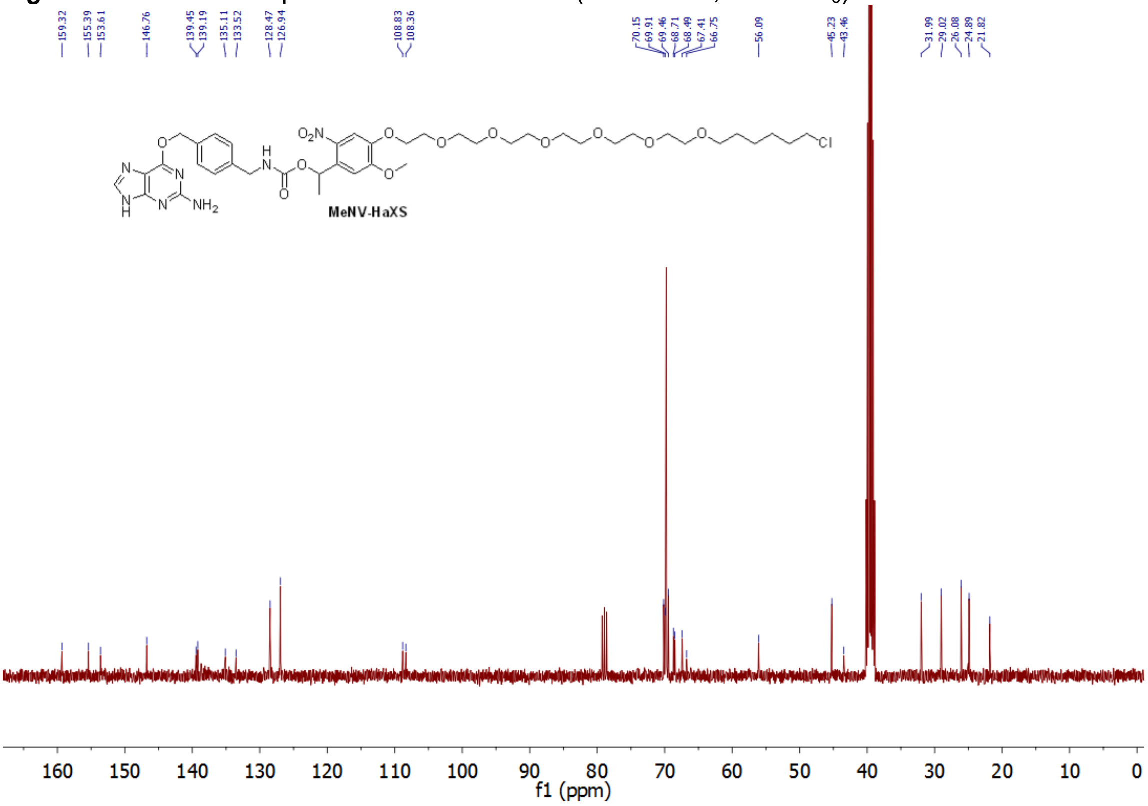

5b. HRMS spectra

Figure S20: HRMS spectrum of **5**

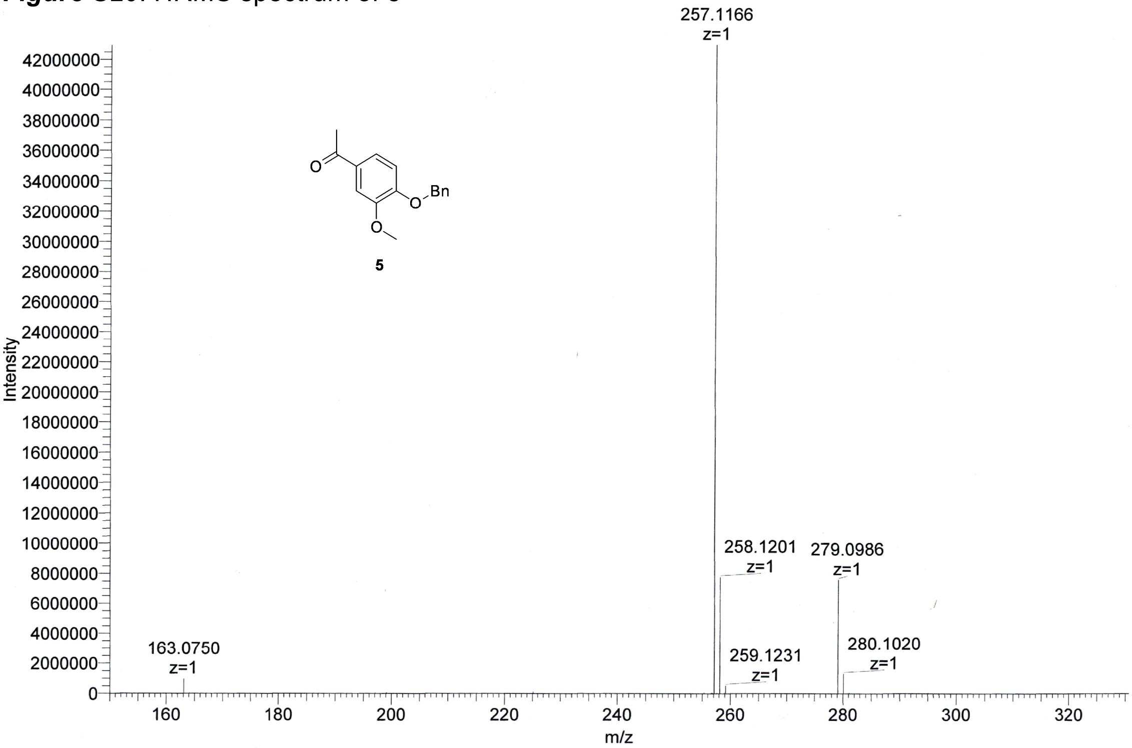

Figure S21: HRMS spectrum of **6**

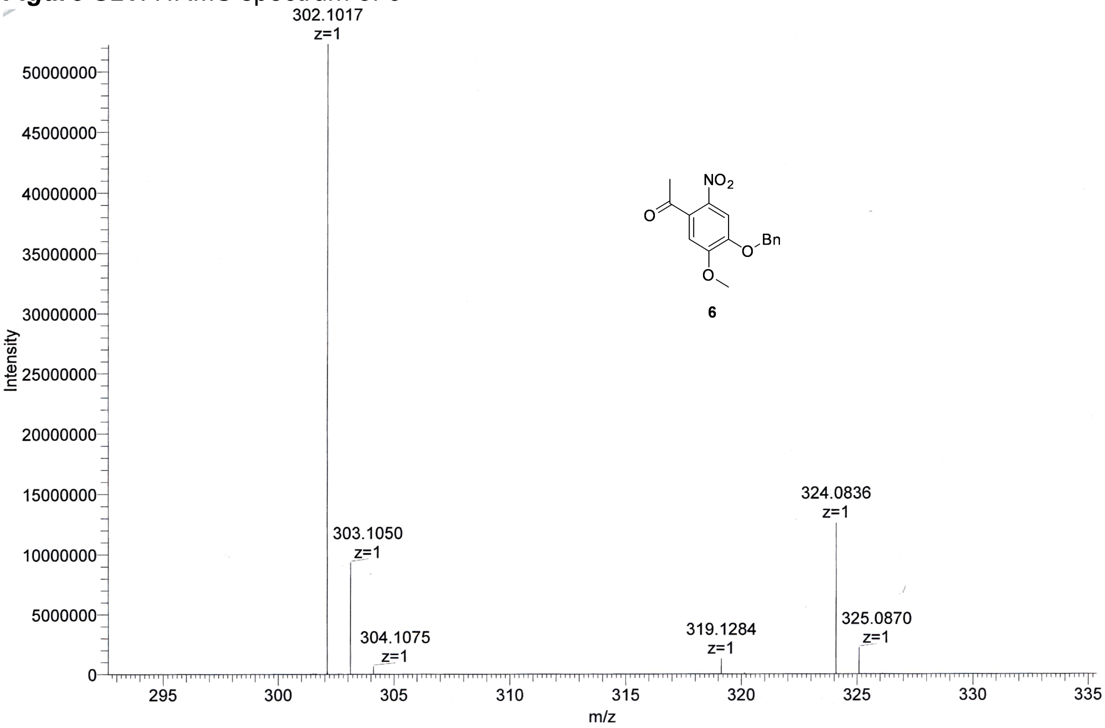

Figure S22: HRMS spectrum of **7**

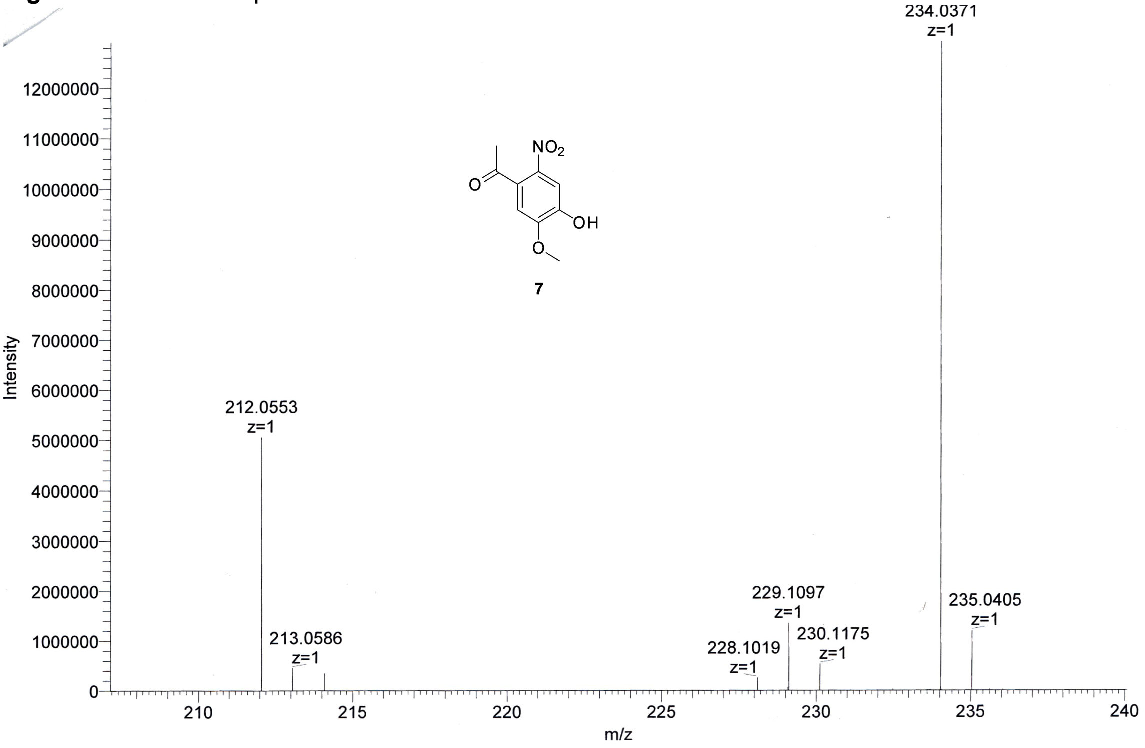

Figure S23: HRMS spectrum of **8**

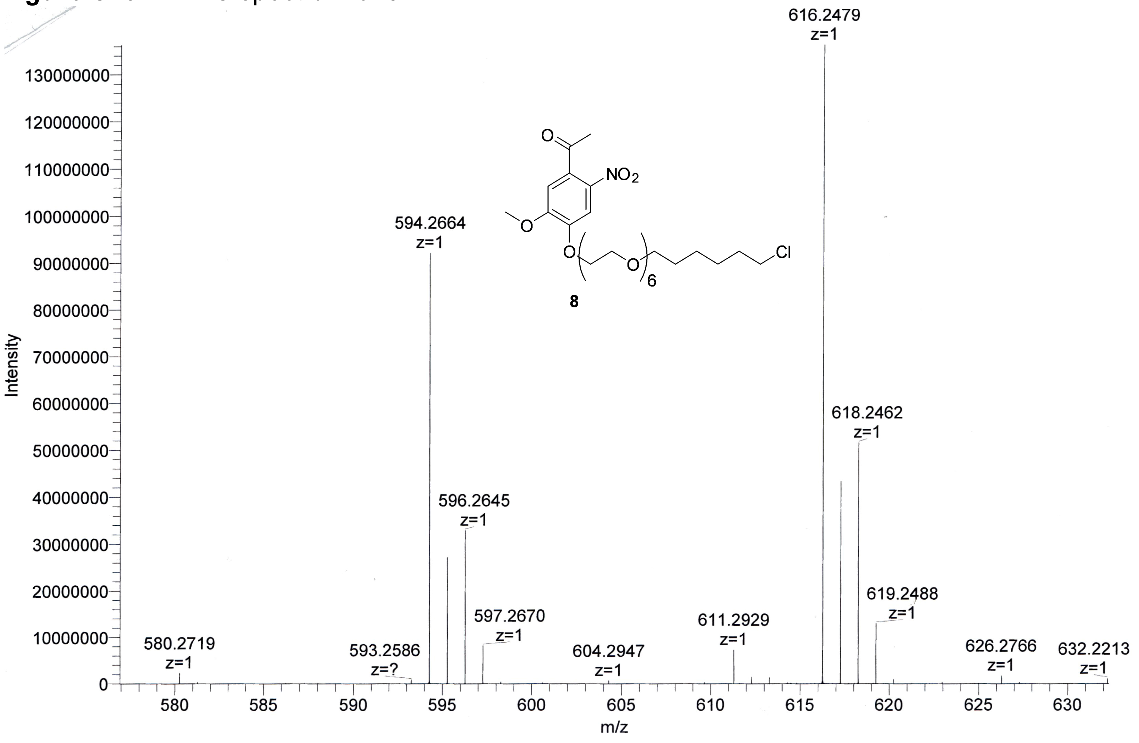

Figure S24: HRMS spectrum of **9**

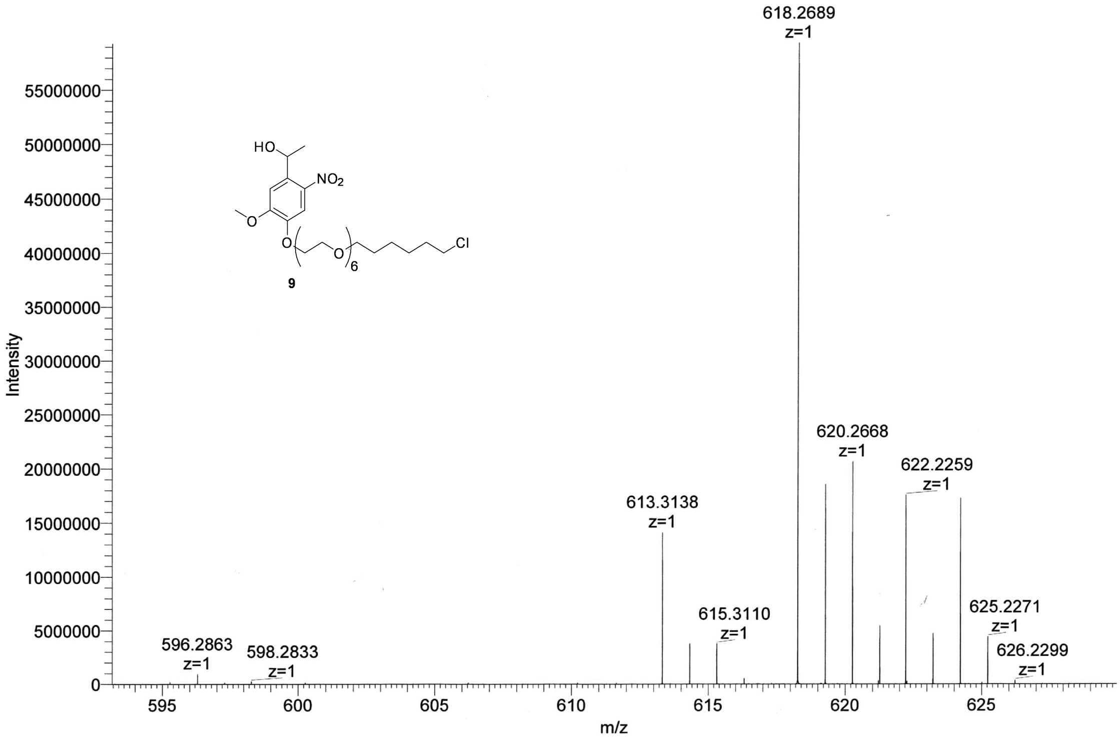

Figure S25: HRMS spectrum of **MeNV-HaXS**

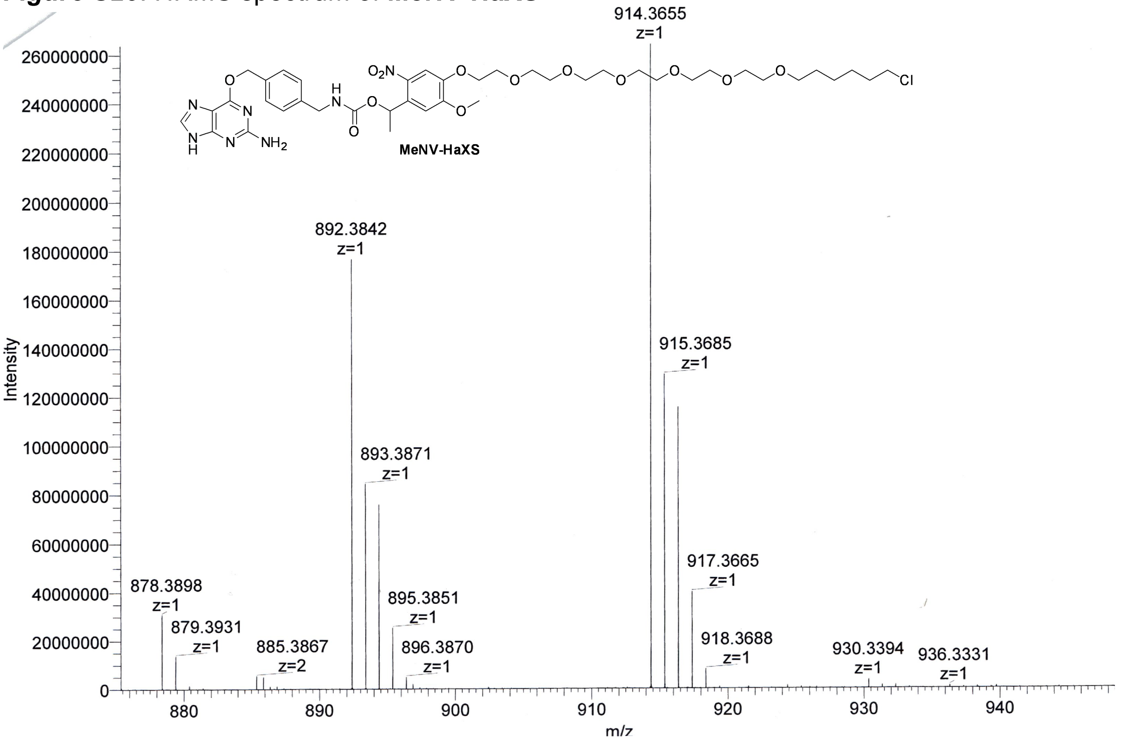

Supplement: Supplementary file 1 [file anie0053-4717-sd1.pdf]
